# Synthetic Micrographs of Bacteria (SyMBac) allows accurate segmentation of bacterial cells using deep neural networks

**DOI:** 10.1186/s12915-022-01453-6

**Published:** 2022-11-30

**Authors:** Georgeos Hardo, Maximilian Noka, Somenath Bakshi

**Affiliations:** Department of Engineering, University of Cambridge, Trumpington Street, Cambridge, UK

**Keywords:** Image analysis, High-throughput imaging, Microfluidics, Timelapse microscopy, Bacterial cell imaging, Deep-learning, Synthetic images, Cell segmentation

## Abstract

**Background:**

Deep-learning–based image segmentation models are required for accurate processing of high-throughput timelapse imaging data of bacterial cells. However, the performance of any such model strictly depends on the quality and quantity of training data, which is difficult to generate for bacterial cell images. Here, we present a novel method of bacterial image segmentation using machine learning models trained with Synthetic Micrographs of Bacteria (SyMBac).

**Results:**

We have developed SyMBac, a tool that allows for rapid, automatic creation of arbitrary amounts of training data, combining detailed models of cell growth, physical interactions, and microscope optics to create synthetic images which closely resemble real micrographs, and is capable of training accurate image segmentation models. The major advantages of our approach are as follows: (1) synthetic training data can be generated virtually instantly and on demand; (2) these synthetic images are accompanied by perfect ground truth positions of cells, meaning no data curation is required; (3) different biological conditions, imaging platforms, and imaging modalities can be rapidly simulated, meaning any change in one’s experimental setup no longer requires the laborious process of manually generating new training data for each change. Deep-learning models trained with SyMBac data are capable of analysing data from various imaging platforms and are robust to drastic changes in cell size and morphology. Our benchmarking results demonstrate that models trained on SyMBac data generate more accurate cell identifications and precise cell masks than those trained on human-annotated data, because the model learns the true position of the cell irrespective of imaging artefacts. We illustrate the approach by analysing the growth and size regulation of bacterial cells during entry and exit from dormancy, which revealed novel insights about the physiological dynamics of cells under various growth conditions.

**Conclusions:**

The SyMBac approach will help to adapt and improve the performance of deep-learning–based image segmentation models for accurate processing of high-throughput timelapse image data.

**Supplementary Information:**

The online version contains supplementary material available at 10.1186/s12915-022-01453-6.

## Background

High-throughput time-resolved imaging of bacterial cells has revolutionised the fields of systems and synthetic microbiology [1–7]. Deep-learning–based approaches are rapidly gaining popularity for automated processing of such high volumes of data [8–13]. The performance of such deep-learning algorithms is fundamentally dependent on the quality and quantity of the training data provided to it [14]. However, the task of generating training data with accurate ground truth is not only slow and difficult but a near-impossible one for images of micron-sized bacterial cells. The comparable size of the point spread function (PSF) of the microscope corrupts the images of bacterial cells, blurring them to the extent that neither expert users nor traditional computational segmentation programs can accurately annotate the correct pixels. Additionally, geometric effects in the microscopic 2D projection of 3D objects in an image lead to inaccuracies in contrast-based segmentation by both humans and computational programs (Additional file 1, Section 1, [15, 16]). Inaccuracies in the ground truth of training data cause deep-learning models to misinterpret the relations between the object and its image, resulting in systematically artefactual mask predictions. This limits one’s ability to infer a cell’s true size and shape and confounds the analysis.

To address these limitations, we have developed a new approach of image segmentation of bacterial cells, where deep-learning networks are trained entirely on synthetic data. The main challenge here is to generate synthetic data that accurately reflects the object-to-image relations in a diverse range of imaging modalities and platforms. To tackle this, we have created SyMBac (*Sy*nthetic *M*icrographs of *Bac*teria), a tool to generate realistic synthetic images of bacteria in a wide range of experimental settings. SyMBac combines detailed models of cell growth and morphology, its position and interaction with the imaging platform, and the microscope’s optics to render synthetic images capable of training highly accurate segmentation networks without human-annotated data. A neural network trained on this synthetic data can precisely learn about the corruption modes introduced during image formation and, as a result, output accurate cell masks. This not only greatly speeds up the process of image segmentation (because no human annotation is necessary), but most importantly generates more accurate masks of cells, enabling precise analysis of size regulation and growth dynamics. Using our method, given any change in experimental settings, it becomes trivial to generate high volumes of new synthetic images and ground truths to retrain segmentation models and rapidly analyse new data. This also addresses the robustness and reproducibility problems of image processing using deep-learning because models can now be easily adapted and benchmarked, since synthetic data can be generated at any desired spatial and temporal resolution with known parameters.

In this paper, we show that SyMBac can be used to generate synthetic micrographs for linear or monolayer colonies of bacteria in microfluidic devices or agar pads. While SyMBac can be easily used with fluorescence images, we primarily focus on the more challenging task of tackling phase-contrast images of bacteria in these platforms. This is because phase-contrast imaging can be used ubiquitously for all microbes since it does not require any kind of labelling and also because it poses a more difficult challenge for segmentation algorithms, as the images contain the structures of the imaging platform, making it difficult to separate them from the cells. This problem is most severely felt in microfluidic linear colonies (mother machine [17]) and single-cell chemostats [18], where the microfluidic device’s features are comparable to the size and refractive index of the cells which they trap, giving them similar contrast. However, microfluidic linear colonies are also of major interest to quantitative microbiologists, as they are able to provide the highest throughput and most precise control of growth conditions [3, 7, 19]. This has recently revolutionised research in quantitative single-cell bacterial physiology [17, 20–23]. Accurate segmentation of individual cells in these platforms is sorely needed for further and deeper investigations into single-cell physiology. Our results show SyMBac can be used to rapidly generate realistic training data for these platforms, which is capable of training accurate segmentation models. Machine learning models trained with SyMBac data is highly accurate in cell identification and produce more precise masks of cells in these platforms compared to models trained on data generated or curated by humans. This enabled precise analysis of cell size and shape regulation along changing conditions in a growing bacterial cell culture, revealing novel insights about the physiological dynamics of individual bacterial cells during entry and exit from dormancy.

## Results

### SyMBac allows for generation of realistic synthetic data in a variety of experimental conditions

**Fig. 1:**
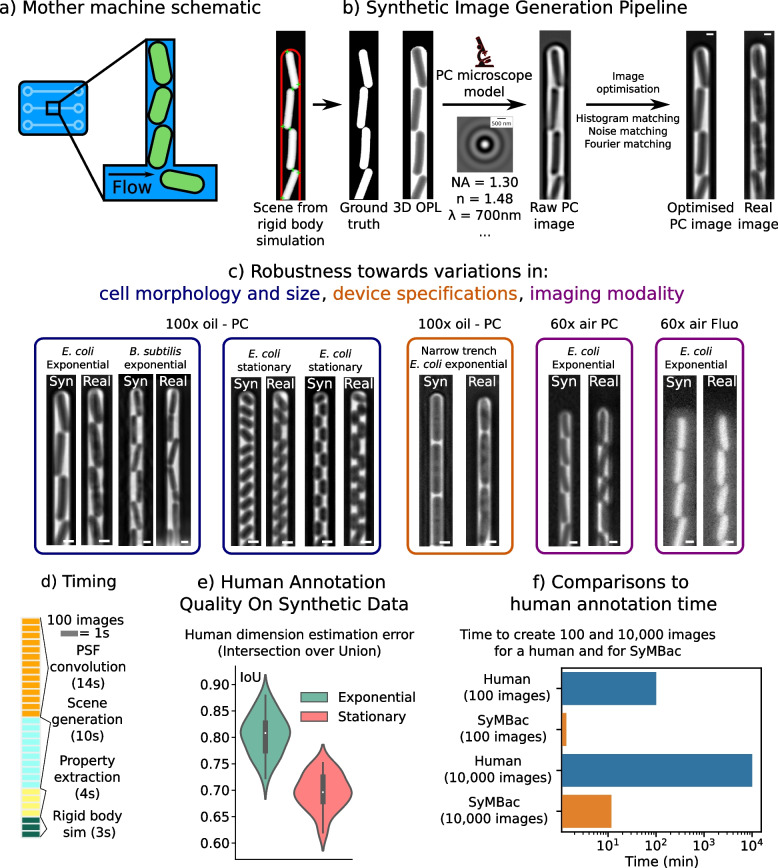
The synthetic image generation process: **a** Schematic of linear colonies of cells in a microfluidic device known colloquially as the mother machine. **b** Synthetic image generation pipeline: rigid body physics simulations are combined with agent-based modelling to simulate bacterial growth in the device. These simulations are convolved with the microscope’s point spread function, which is generated using known parameters of the objective lens. This output image is then further optimised to match real images. Scale bar = 1 μm. **c** Synthetic data can be adapted to different biological conditions, variations in microfluidic designs, and imaging modalities. With real data, many experiments would need to be conducted to generate training data with the same kind of coverage. Scale bar = 1 μm. **d** Typical timescales for individual steps in the generation of training data. **e** Humans annotating images had variable performances and consistently undersegmented cells, especially in small stationary phase cells. **f** SyMBac is approximately 10,000× faster than a human at generating training data (10,000 images in less than 10 min)

In Fig. 1b, we show the overall process of generating synthetic training data for bacterial cells growing in microfluidic linear colonies, which is SyMBac’s primary use in this paper. A rigid body physics simulation is combined with an agent-based model of bacterial cell growth to generate model scenes of cells growing in these devices (details of the model parameters and implementation are provided in Additional file 1, Section 2, [24]). The output position and geometry of this combined model are then used to produce synthetic micrographs and the corresponding ground truths through the following steps. First, properties from the simulation are extracted and used to render an optical path length (OPL) image that signifies the relative phase shift in the image—observed as as a change in intensity—due to the local refractive index (Fig. S3.1 in Additional file 1). To generate micrographs, this OPL image is then convolved with the microscope’s point spread function (PSF) which can be generated using known parameters of the objective lens and phase ring/annulus (Additional file 1, Section 4 [25–27]). These properties are easy to find and thus any change in microscope optics can be readily simulated. SyMBac offers 2D approximations to microscope optics, but also has an option for 3D optical models, allowing the user to trade image accuracy for simulation speed. Next, users have an option to add a camera model (if the user knows the properties of their camera) to simulate the image capture process. Finally, the intensity distribution and noise spectrum of the resultant image are then further optimised in order to maximise its similarity to the real images (Additional file 1, Sections 7 and 8).

**Fig. 2:**
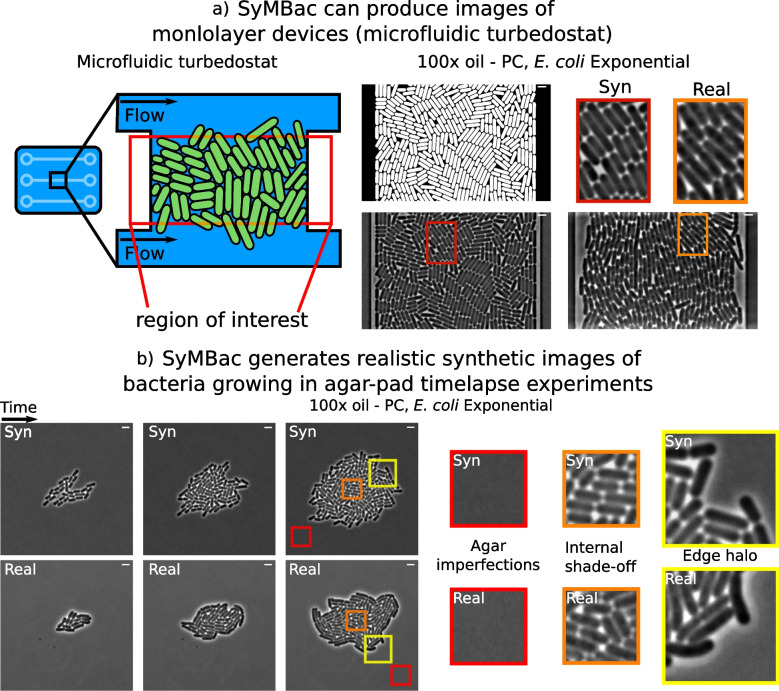
Different synthetic image modalities: **a** Synthetic data can be generated for microfluidic devices that produce monolayer colonies, in this case the microfluidic turbedostat described in [28] (real image courtesy of Elf Lab, Uppsala University). Scale bar = 2 μm **b** SyMBac can also generate timelapse image data for the growth of monolayer colonies on agar pads. Scale bar = 2 μm

Synthetic images generated by SyMBac can be tuned according to variations in biology (cell size, shape, etc.), the imaging platform (microfluidic device architecture, agar pad), optics (magnification, numerical aperture, and additional properties of the objective lens), and whether the experiment is phase contrast or fluorescence (Fig. 1c). The physics back-end of SyMBac can be adapted to model alternative imaging platforms, to generate synthetic training data for imaging experiments of both 1D and 2D colonies (Additional file 1, Section 13). Put together, this enables us to produce realistic phase-contrast and fluorescence images of bacterial cells in microfluidic platforms and on agar pads (Figs. 1c, 2, and 6). In Fig. 2a, we demonstrate the use of SyMBac to generate synthetic phase-contrast images for the microfluidic turbidostat devices described in [28]. Comparison of the close-ups from the synthetic image and the real image from such devices reveals the similarities in texture and contrast, which are crucial to ensure that the synthetic training data is realistic enough for models trained with it to perform well on real data (as shown in Fig. 6). Similarly, it is possible to generate synthetic micrographs of timelapse experiments of the growth of monolayer colonies on agar pads. The close-ups of the synthetic images show that the model captures all the relevant details: background texture from agar imperfections, contrast shade-off effects near the centre of the monolayer colony, and background halo and darker cells near the edge of the colony (Fig. 2b and Additional file 1, Section 13).

SyMBac data also provides an excellent opportunity for benchmarking the performance, speed, and accuracy of human annotators and traditional thresholding-based computational segmentation approaches, as perfect ground truth is available for each image (Additional file 1, Section 1). Our benchmarking experiments show that human annotators are not only slow, but generated inaccurate training data (Fig. 1f, e), being unable to maintain consistency or accuracy in correctly identifying pixels at the cell boundary. Additionally, we found that annotation results were highly variable across growth regimes, with the error rate in annotating small cells from the stationary phase being higher (Additional file 1, Section 1). While better than human-annotated training data, we also find that the unsupervised approaches using Otsu, local thresholding, and membrane dye images still suffer from bias and lack of robustness towards size variation (Figs. S1.2, S1.3, and S1.4, in Additional file 1). This suggests that these traditional approaches are not suitable when accurate cell masks are desired and would not be suitable for generating training data required for training ML algorithms to accurately segment data. SyMBac addresses this critical issue, since the synthetic images from SyMBac are accompanied with perfect ground truth information of cell shape and position. Models trained on our data therefore ensure the neural network learns the true relation between object and its corresponding image.

SyMBac takes advantage of multiprocessing, and where possible offloads array operations onto the GPU, which allows it to be approximately 10,000× faster than a human at generating training data (and if deployed on computing clusters, this could be extended by a further order of magnitude). Typical timescales of generating synthetic images are shown in Fig. 1d. The entire process of generating synthetic data has been made simple through the creation of example Jupyter notebooks with interactive widgets, which guide the user through the entire process. The output format of the training data (synthetic images and ground truth) can be chosen as single frames or tiles, depending on the segmentation network of choice. We provide example notebooks of training deep-learning segmentation networks, such as DeLTA and Omnipose with SyMBac data, and notebooks for using one’s trained models for image segmentation. This information is kept up to date online through browsable documentation.

### SyMBac enables easy training of existing deep-learning segmentation models

**Fig. 3:**
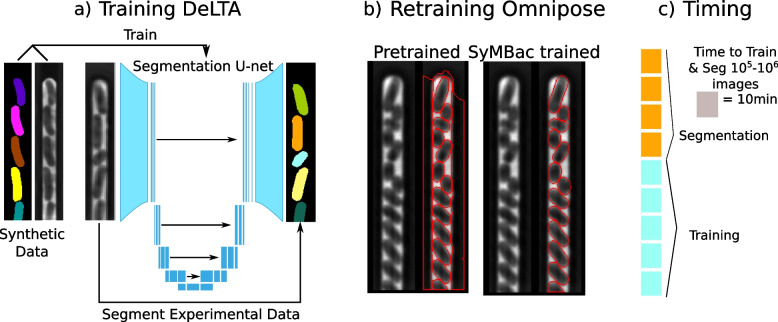
Model training, evaluation, and timing benchmarks: **a** Schematic of the U-net model being trained using synthetic data and then segmenting real data to produce accurate masks. **b** SyMBac can retrain generalised models, such as Omnipose (a derivative of Cellpose, allowing for mask reconstruction from arbitrary morphologies). Because Omnipose was not trained on any microfluidic device images, it fails to properly segment the image, attaching masks to the mother machine trench geometry (though it admirably segments cells within the trench). Retraining Omnipose with SyMBac’s synthetic data results in near perfect segmentation, with no more trench artefacts. **c** A typical time to train the network, either Omnipose or DeLTA (on 2000 images) and segment approximately one million images (Nvidia GeForce 1080Ti)

Once synthetic images are generated, a segmentation network (e.g. U-net [29]) is trained. This network learns the relation between the synthetic image and ground truth pair to infer image-to-object relationships in real experimental images (Fig. 3a). We evaluated the usefulness of synthetic data for this purpose by applying it two popular methods: (1) DeLTA, an implementation of U-net [30] for analysing images of cells in linear colonies, and (2) OmniPose [31], a deep neural network for morphologically robust segmentation of bacteria. For DeLTA, since the output from the U-net is only a probabilistic mask, a correct probability threshold must be determined to compute accurate binary masks. To calculate the optimal probabilistic threshold value, we developed a validation mechanism which utilises synthetic data to estimate the optimal mask pixel probability threshold by passing an independent set of synthetic validation data through the trained model and thresholding the output until the masks are maximally similar to the ground truth masks. Two metrics can be used to evaluate the optimal mask threshold, one is the Jaccard index, which is a pixelwise measure of the difference between the true masks and the target thresholded masks. The other measure involves maximising the cumulative intersection between the length and width distributions of cells in the true masks with those in the target thresholded masks. We show that, for these experiments, these independent metrics produce the same optimal mask threshold (Fig. S9.1a in Additional file 1). Omnipose, the second network we tested, does not require any thresholding of the masks by the user. Additionally, training data for Omnipose differs from training data for DeLTA in that cell masks are allowed to touch in the Omnipose model, whereas DeLTA requires the computation of weightmaps around the edges of cells. Omnipose is therefore easier to train, output masks require no thresholding, and masks are allowed to touch, as Omnipose returns an instance segmentation. While the published pretrained Omnipose model is already robust for most applications, it is unsuitable for use with microfluidic devices and produces artefacts (Fig. 3b). We retrained Omnipose with SyMBac data and the resultant model produced morphologically accurate masks of cells in microfluidic devices and still maintained good performance in other imaging platforms.

Since SyMBac uses synthetic data for training, validation data is not available in the traditional sense. Instead, the model is evaluated automatically epoch-by-epoch in terms of cell identification errors, and the model with the lowest error is kept (Fig. S9.1b in Additional file 1). Automatic identification error rate calculations are possible without validation data due to the predictable nature of bacterial growth in linear colonies (detailed in Additional file 1, Section 9). In general, 100 epochs were enough to drive the identification error below $0.5\%$ with DeLTA. Up to 400 epochs were necessary to achieve less than $0.2\%$ error. The Omnipose model was trained for 4000 epochs and was able to achieve $<0.1\%$ error. Typical timescales for model training and segmentation are shown in Fig. 3c.

### Models trained on synthetic data show superior performance and precision compared to those trained on human-annotated data

**Fig. 4:**
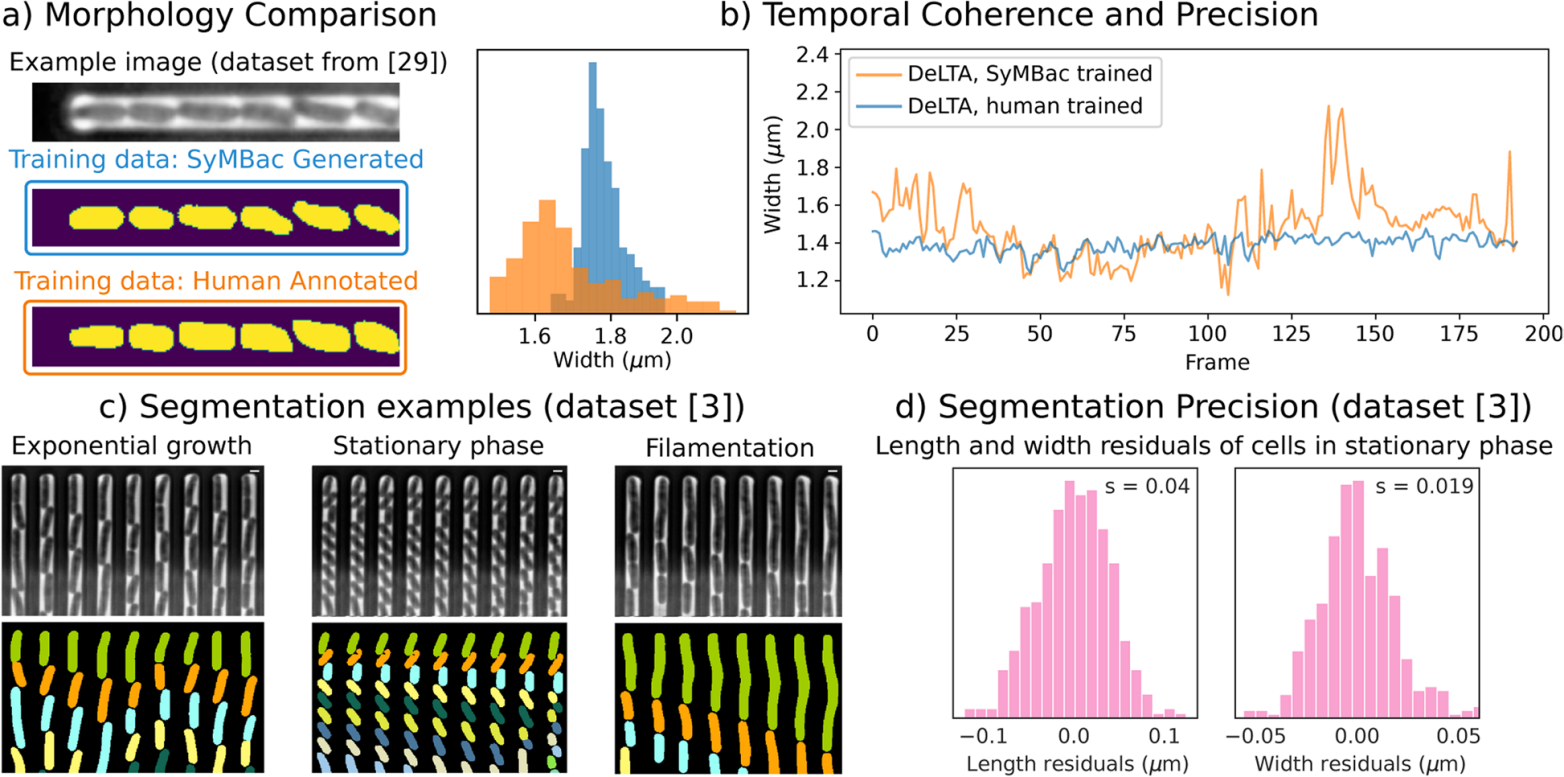
Model quality and segmentation precision: **a** The masks from SyMBac-trained models are truer to the geometry of the cells, displaying no aberration when compared to model outputs trained on human-annotated data. **b** The masks also maintain a narrow distribution of widths, while the masks from DeLTA trained with human-annotated data display a wide variation with the peak shifted to lower values and show 2.5× higher variation in cell width. **c** Examples of the type of data which can be segmented using a single SyMBac-trained model. In this case, we show the performance of a single DeLTA model trained on combination data across 3 different cell sizes. Scale bar = 2 μm **d** The SyMBac-trained model produces masks with precisions of 40 nm for length and 19 nm for width. This is calculated by fitting a line to the length and width trace of cells in the stationary phase

SyMBac-trained models produce more consistent, less flawed, and visually more “natural” cell mask shapes compared to models trained with user-annotated data (Fig. 4a, b). This is because masks from synthetic data are never corrupted by changes in the images from which they come and are accompanied with perfect ground truth. To perform a rigorous comparison between synthetic data and human-generated data, we generated a synthetic dataset based on reference images from the original DeLTA paper’s dataset using SyMBac. We then trained two models, one on SyMBac data and one on the training data provided in the DeLTA paper [30]. In the DeLTA paper, a semi-automatic tool was used to generate training data, which involves subjectivity in the choice of thresholding parameters and morphological operators. This results in training data with oddly shaped masks, which are consequently learned and then reproduced by the network during training and prediction (Fig. 4a, Additional file 1, Part 10). The networks trained with SyMBac produce far more realistic and consistent mask predictions compared with models trained with human-curated training data (dataset from Lugagne et al. [30]) while maintaining almost the same accuracy (Table 1). Furthermore, we observe that the DeLTA model trained with SyMBac shows much higher temporal coherence when compared to models trained on human-annotated data. Output masks from the DeLTA model trained with human-curated training data showed unrealistic fluctuations in cell width from frame to frame, while SyMBac-trained models did not (Fig. 4b).

**Table 1:** Performance (cell identification error) benchmarking of DelTA trained with SyMBac. Models trained with SyMBac perform well for both oil and air objectives and outperform models trained with user-generated training data

Dataset	DeLTA (pretrained)	DeLTA (SyMBac trained)	Omnipose (SyMBac trained)
100×, oil, NA 1.45, [30]	0.93%	1.1%	0.25%
100×, oil, NA 1.49 [3]	1.8%	0.47%	0.12%
60×, air, NA 0.95 [3]	N/A	0.35%	0.28%

DeLTA models trained with SyMBac data were able to achieve comparable identification accuracy (table in Table 1 as the model trained on human-annotated and human-curated data, which is remarkable, since it shows that SyMBac is able to generate realistic training data from just the reference images. Notably, the true advantage of SyMBac is in its ability to generalise to experimental variations where pretrained models fail. When we tested against a data set collected with a 60× air objective, the pretrained DeLTA model could not produce any masks, and therefore, we could not quantify the error. Conversely, SyMBac can generate similar images and ground truth pairs, and DeLTA models trained with this data produced highly accurate segmentation with an identification error rate of $0.36\%$ (Table 1) and high-quality cell masks (examples in Fig. 4c and Additional file 1, Section 11). Precise segmentation of images from low NA air objective has been a major challenge, and this capability opens up possibilities of analysing high-throughput experiments conducted with such objectives, due to their large field of view and fast scan speed. DeLTA trained with SyMBac data also gave 18× better identification accuracy compared to pretrained DeLTA models for a separate 100× dataset (experiments detailed in Bakshi et al. [3]). Interestingly, Omnipose models trained with SyMBac produced higher identification accuracies under all imaging conditions tested here. The possible explanations are Omnipose’s robustness to morphological variations and being able to segment very closely packed cells due to its mask reconstruction algorithm. Omnipose models trained on SyMBac data also yielded much more realistic cell masks compared to Omnipose models trained on human-annotated training data (dataset from Lugagne et al. [30]).

### Models trained with synthetic data are robust to changes in experimental conditions

**Fig. 5:**
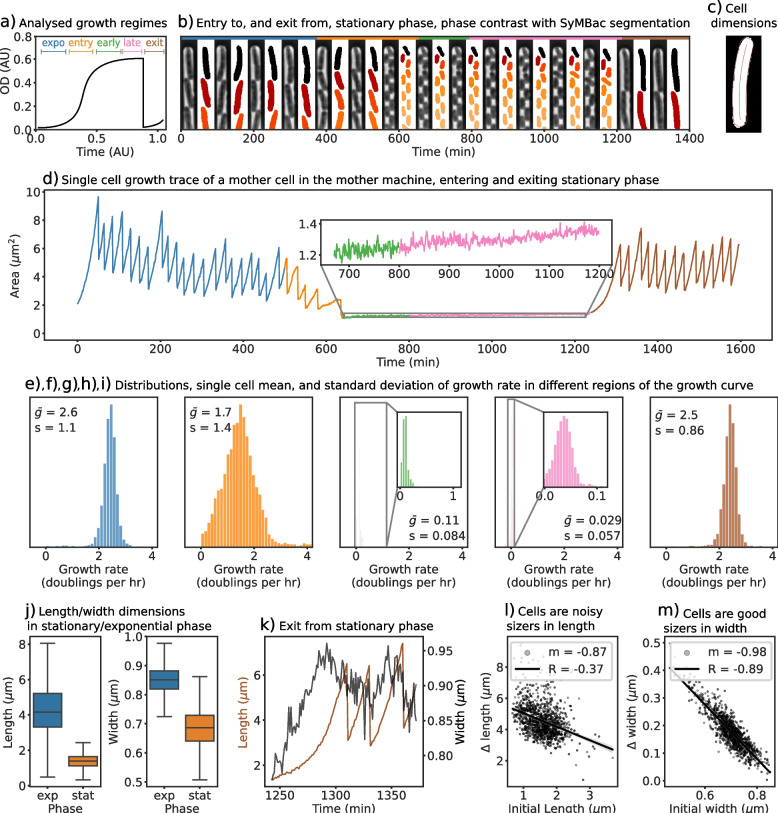
**a** Models trained with SyMBac were used to segment single-cell data throughout all growth curve regimes (colour-coded and used throughout the figure). **b** Example kympgraphs of 100× data showing cells in a variety of states (exponential growth, stationary phase, filamenting) with accompanying masks, highlighting the robustness of the model trained on mixed data to segment cells of multiple cell sizes and morphologies. **c** An example output showing the coordinate system applied to a cell mask, generated by Colicoords [16], allowing for highly accurate length and width prediction. **d** Example time series of the size of a single cell going through an entire growth curve. The inset shows cell length changes during the stationary phase. **e**–**i** During the exponential phase, cells exponentially increase their size with a mean growth rate of 2.6 volume doublings per hour, which is equivalent to a population doubling time of 23 min, consistent with the bulk growth measurements of cells in this richly defined medium [3]. The distribution of growth rates shifts to the left as cells enter the stationary phase (orange and green phase) and eventually stops 6 h into the stationary phase (pink). For all growth rates, corresponding standard deviations are also reported. **j** Cells show a wide distribution of lengths during the exponential phase which narrows greatly during entry to the stationary phase, as cells are “locked in” to their width. Interestingly, while the mean width decreases in the stationary phase, the variability in cell widths increases. **k** Example of a cell exiting stationary phase, showing the increase in length and width. **l** Comparison of initial length and the added length before the first division after exit from stationary phase shows that cells are noisy as sizers towards length regulation. **m** Comparison of the initial width and the added width before the division shows that *E. coli* is an almost perfect width-sizer, dividing only when individual cells reach a critical width

Segmentation algorithms (both ML and non-ML) face an even harder challenge when the data type itself changes during the experiment, for example, cells changing size, shape, and intensity along a growth-curve experiment as they transition from exponential to stationary phase and back (Fig. 5a) [3]. Since cells in the stationary phase are very small, the relative annotation error grows (as shown in our benchmarking results in Fig. S1.1 in Additional file 1) and compromises the performance of the ML algorithm. On the other hand, non-ML methods suffer from changes in object size, as algorithmic parameters need to be retuned when large changes are observed in the image (for example, morphological operators need to have their window size changed to account for differently sized cells). SyMBac can address this issue by generating high-quality training datasets for cells at any size (Fig. 1c), producing accurate cell masks throughout the experiment (Fig. 4c and Fig. S11.2 in Additional file 1). While models trained with small cell training data perform well in the stationary phase, this comes at the cost of reduced accuracy in the exponential phase, and the converse is also true (table in Table 2); however, this effect is much less pronounced when training Omnipose models. Instead, a complete dataset, which combines synthetic images of both types, leads to an overall better performance in identification accuracy and highly precise masks for estimating cell length and width (Table 2). The models trained on combination datasets are robust to large variations in cell size (Additional file 1, Section 11), while maintaining the ability to detect very small variations in size due to the segmentation precision. This opens up possibilities of deeper investigations into cell size and shape regulation, as cell width can now be accurately characterised.

**Table 2:** Comparison of DeLTA and Omnipose trained on 3 types of synthetic training data: data containing large cells only, data containing small cells only, and the combination of these two cell types to produce a combined dataset. As expected, specialist models tend to perform better when segmenting their own data type, and worse when segmenting unseen data types. A model trained on a combination of the two datasets provides a good compromise, not needing to train multiple models, while retaining good performance across the entire dataset. Interestingly, the Omnipose model trained on combination data performed as well on exponentially growing cells as the same model trained only on large cell synthetic data, implying that the network has residual capacity to learn. This was also noted in the original Cellpose paper [32]

Dataset	Model: DeLTA			Model: Omnipose		
	Training data			Training data		
	Large	Small	Combined	Large	Small	Combined
Exponential [3]	0.10%	2.4%	0.14%	0.09%	0.37%	0.09%
Stationary [3]	1.6%	0.40%	0.80%	0.89%	0.12%	0.20%
Full growth curve [3]	0.95%	1.4%	0.47%	0.45%	0.25%	0.12%

### Precision of synthetic data trained models reveals novel width regulation in *E. coli* exiting stationary phase

As a proof-of-principle application, we have used SyMBac-trained models to analyse a timelapse experiment (1800 frames) of 4500 mother cells growing in linear colonies under changing growth conditions, whereby the cells undergo a feast famine cycle [3]. The segmentation network maintained high identification accuracy and produced visually accurate masks of cells throughout the experiment (Fig. 4c). To estimate the size and shape of individual cells from these accurate masks, we used colicoords, a python package for generating cell coordinates from segmentation outputs ([16]).

The precise estimates of cell size from the segmentation outputs enabled us to compute single-cell growth rates during the exponential phase, along the entry and exit from the stationary phase along a growth curve (Fig. 5d), and at different points within the stationary phase (Fig. 5e–i). During exponential growth, cells elongate at a mean rate of 2.6 volume doublings per hour, which drops to 1.7 volume doublings per hour as cells begin to enter the stationary phase, and then asymptotically decreases as cells progress deeper into the stationary phase. Notably, a small residual, but detectable growth rate is maintained (0.029 volume doublings per hour), which is approximately 100 times slower than the exponential growth rate (Fig. 5f–h). When fresh media are introduced to the deep stationary phase cell culture, cells recover their growth rates as they re-enter exponential growth (Fig. 5i). Since the cells almost stop growing in the deep stationary phase, the fluctuations in the cell size estimates from the binary mask can be used to estimate the precision of segmentation (Fig. S9.5 in Additional file 1). We estimated the precision of the output from SyMBac models to be 40 nm along the length and 19 nm along the width (Fig. 4d).

The highly precise estimates of cell size and shape enable us to look at shape regulation at different stages of starvation and resuscitation. During the entry to the stationary phase, both the length and width of individual cells drop. The large variability of cell lengths during the exponential phase is a result of exponential growth between division events, but cell width is tightly regulated in this phase (Fig. 5j). During resuscitation, cells increase their width and length and divide once they have reached a critical size (Additional file 1, Section 12). Upon further investigation, we find that cells are sizers in both width and length during exit from the stationary phase. Interestingly, cells seem to more strictly regulate their width than their length during wake up and each cell divides upon reaching a critical width (Fig. 5l vs m). Tight regulation of width for size control makes intuitive sense, as the volume of a cell has a stronger dependence on width (radius) than length ($V = \pi r^2(l - \frac{2}{3}r)$). The large variability in cell width in the stationary phase coupled with the strict width requirement for initiating cell division leads to a strong correlation between the shape and size of individual cells and their resuscitation behaviour, with important implications for persistence towards antibiotics, and population fitness in general.

### SyMBac can be used to train accurate segmentation models for various imaging platforms and modalities

**Fig. 6:**
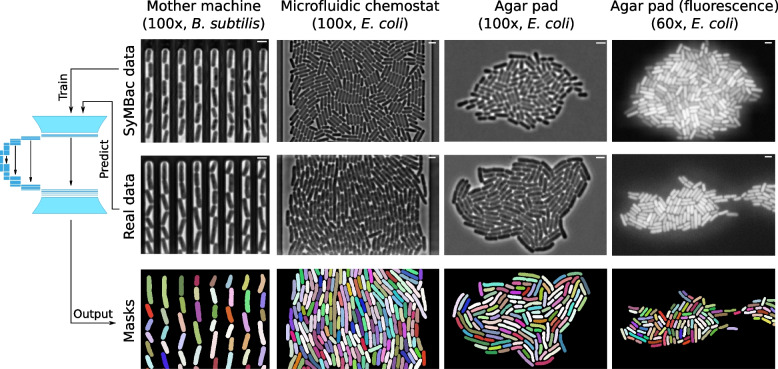
Extensions of SyMBac for cell segmentation in images of linear colonies of *B. subtilis* (very straight cells, unlike *E. coli* which have more curvature), monolayer colonies in a 2D microfluidic turbidostat chamber (data from [28], kindly provided by the Elf Lab, Uppsala University), growing colonies on agar pad, and low-resolution fluorescence snapshots of dense cell clusters on agar pad. Scale bar = 2 μm

Finally, while we demonstrated that SyMBac is most powerful in its ability to train highly accurate models for image segmentation of cells growing in linear colonies, we also sought to extend SyMBac to produce synthetic micrographs of cells growing in monolayer colonies, such as the “biopixel” device [33], 2D turbidostat [28], and even colonies growing on agar pads. Models trained with this data (DeLTA models) produced accurate segmentation of test images (Fig. 6) (image data generously provided by the Elf Lab, Uppsala University). To extend the usability of SyMBac to timelapse images collected on agar pads, we have also simulated the growth of micro-colonies in both fluorescence and phase contrast and generated corresponding images (Fig. 6) which are very similar to real micrographs. Modifications to the simulation, the point spread function, and the segmentation pipeline are described in Additional file 1, Section 13. These synthetic images were used to train deep-learning networks to achieve good segmentation of experimental images from corresponding test images and produced masks of unprecedented qualities, in terms of the shape of individual cells and robustness towards the variation in size and contrast.

## Discussion

Here, we have demonstrated a new approach of cell segmentation using deep-learning models trained entirely on synthetic training data. Synthetic data has been used previously to aid in segmentation. MiSic [34] is a tool for segmenting bacterial micrographs based on real training data, but supplements this data with “synthetic” data, which however does not accurately reflect the optics, physics, or biology of the experiment which is being analysed, and thus cannot segment images based purely on synthetic data. BCM3D [35] is a tool used for fluorescence biofilm segmentation; however, (1) it is not compatible with linear and monolayer colonies in microfluidic devices that are typically used in high-throughput quantitative experiments, and (2) its synthetic data is only generated in fluorescence and not compatible with phase-contrast images, which are ubiquitous in the imaging of bacteria. SyMBac is the first tool which can generate realistic synthetic data by modelling the entire experimental setup, end-to-end, and is compatible with phase-contrast and fluorescence images from various imaging platforms, such as microfluidic devices and agar pads. This will provide breakthrough advantages for training segmentation models for accurate performance in various types of imaging experiments.

Training data generated in more traditional ways, using various thresholding algorithms, results in variable masks for cells of identical shape and size when the cell’s pixels have different brightness, illumination profiles, etc. (the same cell would be assigned a different training mask depending on the preprocessing algorithms and parameters used). This introduces uncertainties in the assignment of “true pixels” for each cell, which is passed on to the model from training data and is propagated to the output. On the other hand, while input training masks need preprocessing, so do the output masks [12, 13, 30]. Post-processing these outputs will deform the already artifactual cell masks even more and make cell morphology analysis highly susceptible to error (both systematic and random). Since SyMBac data is accompanied by perfect ground truth information, the resultant masks are highly precise and accurate and do not need any post-processing. This eliminates user subjectivity in data creation, curation, and final analysis, increasing reproducibility. The highly accurate masks from models trained with SyMBac have revealed novel insights into cellular growth and physiology under changing conditions and pave the way for systems-level analysis of the underlying factors using such high-throughput data.

## Conclusions

In conclusion, SyMBac provides a major technical advancement for the easy adoption and robust implementation of machine learning approaches for analysing microscopy data of microbes. Through the rapid and easy generation of high-quality synthetic data with large coverage of experimental setups and cellular morphologies, SyMBac addresses a critical obstacle in the application of machine learning tools for high-throughput single-cell image analysis from various platforms and organisms. Beyond the compatibility towards variations in cell morphology and imaging platform designs, the microscope model in the pipeline is compatible with objectives of various resolutions, including low-resolution air objectives. Therefore, SyMBac enables easy creation of training data for low-resolution microscopy images, which is near-impossible for human annotators or contrast-based computational programs. This enables high-accuracy segmentation from images collected with air objectives (Fig. S11.3 in Additional file 1), which will have important implications in analysing high-throughput image-based screening experiments ([3, 36–38]), where low-resolution air objectives provide the necessary travel distance, speed, and large fields of view. As SyMBac enables the easy creation of synthetic training data at any spatiotemporal resolution, it also addresses the robustness and reproducibility problems with benchmarking machine learning (ML) and non-ML approaches for cell segmentation and tracking. Therefore, we believe SyMBac will play a critical role in improving the performance of the ML algorithms, while also enabling accurate assessments of their performance limits.

## Methods

### Synthetic image generation

**Fig. 7:**
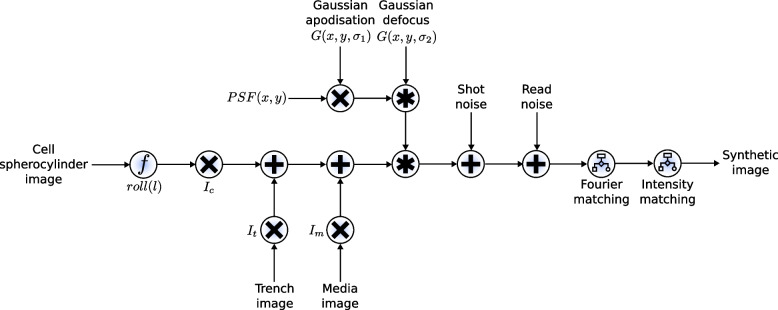
Block diagram of the image generation pipeline. The cell spherocylinder image is first morphed using the roll function and multiplied by $I_c$, the empirical cell intensity. To this image is added the trench OPL image, which is multiplied by $I_t$, the empirical trench intensity. Finally, the media image is added with those pixels being multiplied by $I_m$, the empirical media intensity. These steps are described in detail in Additional file 1, Section 3. The PSF, which has been altered by Gaussian apodisation, and simulated defocus is then convolved over the image. The precise implementation of the PSF and its modifications are described in Additional file 1, Section 4. The camera-modelled shot noise and read noise are then added to the image (implementation details in Additional file 1, Section 6 [39], with optional Fourier and intensity matching occurring after (Additional file 1, Section 7, [40]). Combined, this produces a synthetic image realistic enough to train highly accurate models for image segmentation of real data

#### Simulation of growth and division of cells and their interactions with each other and the microfluidic device

We built an agent-based model (ABM) of cell growth and division, taking into account the three size regulation modes of bacteria (adder, sizer, timer), while also adding variability to the key parameters governing cell division. Cells are agents with properties such as length, width, position, and age, and can be customised to include new properties such as time-dependent fluorescence intensity. These cell agents exist as dynamic objects in a rigid body physics world called a “scene”, modelled using Python bindings of the popular physics engine Chipmunk (pymunk) [24]. Static objects could be added to these scenes which are shaped into microfluidic device geometries, with configurable dimensions. A global event loop keeps track of cell growth, divisions, inter-cellular collisions, and cell-device collisions at each time step. A simple API was created to allow simulations to be run by entering the desired distributions of cell lengths and widths by defining the mean and variance of a cell’s maximum length and average width and then selecting the desired trench geometry (Additional file 1, Section 2). We also incorporate a cell’s probability to lyse at each timepoint, which can be used to simulate antibiotic or bacteriophage susceptibility experiments. The simulation can also be watched in real time to check that the chosen parameters are valid (Fig. S2.1 in Additional file 1).

#### Extracting geometric information and rendering images of the bacteria and device in the scene

Once model scenes are generated from the simulations, geometric information about the cell and trench positions is extracted. This information is the position of each cell’s vertices in every timepoint. The cell’s 3D structure is a spherocylinder, which affects how light interacts with it, yet the simulation engine runs only in 2D. Therefore, to render 3D cells, the cell’s vertices are revolved along their major axis to generate each cell’s spherocylindrical hull. All of these 3D cells are then morphed to simulate cell bending and then re-rendered in a 3D scene (Fig. S3.2 in Additional file 1). A 2D projection of this 3D array is then taken, such that an orthographic projection image is generated where the intensity in each pixel is proportional to the optical path length (OPL) in that region (Fig. S3.1 in Additional file 1). The details of this calculation are provided in Additional file 1, Section 3. This simulated image corresponds to what would be seen if a microscope had no PSF, and thus induced no diffraction artefacts in an image. From the simulated images at this stage, we also save the ground truth images of the cell positions, which act as the masks in our training data.

#### Microscope model for generating phase-contrast and fluorescence images

The microscope model generates images from the orthographic projections by convolving them with PSFs of relevant optics. First, the raw image is rendered at a high resolution (typically 3× the real pixel size) and then convolved with either a fluorescence of phase-contrast PSF, which is also rendered at a higher resolution. After PSF convolution, the image is sub-sampled back to the original resolution. Convolution at higher resolution is more accurate, because sub-resolution features of the PSF (such as the concentric rings) are maintained, and propagated to the image; otherwise, the PSF convolution results would be corrupted by low-resolution artefacts. The user can select an ideal fluorescence PSF or phase-contrast PSF which is modelled as an Airy disk and obscured Airy disk [25] respectively (Additional file 1, Section 4). Additionally, a 3D fluorescence PSF can also be selected for full 3D convolution of the cell volumes. The 3D PSF is provided based on the model given by [41]. A comparison between these two methods is given in Additional file 1, Section 5. The PSFs need to be parameterised by inputting the microscope objective’s numerical aperture, refractive index of the medium, emission wavelength, camera pixel size, and phase-contrast ring and annulus dimensions (details provided in Additional file 1, Section 4).

#### Image optimisation

Next, further optimisation can be used to maximise the similarity between the synthetic image and an example real microscope image. Multiple image adjustments are possible, including intensity histogram matching, noise matching, rotational Fourier spectrum matching, camera noise simulation, and even manual intensity corrections. This is done using an interactive IPython Notebook interface, where the user is presented with a side-by-side comparison of a synthetic image and a real image from their experiment. Two plots showing the relative errors in intensity and variance between cells, the mother machine device, and the space between cells are given so that the user can optimise their image to minimise this error (Additional file 1, Section 7 and Fig. S7.1 in Additional file 1) (Note: While examples of black box optimisers for image similarity matching are included, we avoid their use for this step due to the very noisy error landscape between synthetic and real images. Moreover, we were unable to define a perfect objective function which guarantees perfect image similarity). Once the optimal parameters are identified, large volumes of synthetic images are then generated accordingly. These parameters can also be varied according to a uniform or Gaussian distribution during the image generation process to simulate minor fluctuations in image formation during the experiment. This also acts as a form of data augmentation, but occurs during the image generation process, which preserves the mapping between object and image. The full image generation process is summarised in block diagram form in Fig. 7, and other steps are summarised below, with all implementation details in the supplementary information.

After image optimisation, training samples are generated according to the optimised parameters. We however can simulate uncertainty in an optical system by sampling random deviations in these parameters, such as the level of defocus, the apodisation, and noise levels. This is not data augmentation as it is traditionally understood, as we are simulating mechanistic changes in the image formation process. For the training data generated in this paper, we uniformly sampled ±5% around the chosen parameters.

#### Extension to 2D growth

We have extended the use of SyMBac beyond experiments with microfluidic linear colonies, to include growing monolayer colonies in microfluidic devices and agar pads. For this purpose, the cell simulator back-end was switched from our custom implementation (which is optimised for 1D growth in linear colonies) to CellModeller [42], a more general cell simulator for 2D colonies. Scenes are redrawn in the same way as previously described, and either phase-contrast or fluorescence PSFs are convolved to generate respective image types. The main difference here is the addition of additional phase objects to simulate other microfluidic geometries (shown in Fig. 6), and pseudorandom (Perlin [43]) noise to simulate the textures seen in typical phase-contrast images of microcolonies on agar pads (Fig. S13.3 in Additional file 1). Additionally, the point spread function was adjusted by adding a very small constant offset to modulate the amount of shade-off and halo effects which are characteristic of phase-contrast images (Fig. S13.2 in Additional file 1). The details of this process are described in Additional file 1, Section 13. For 2D geometries, on average, 20 CellModeller simulations were run starting from a single cell, generating in total approximately 1000 unique cell images. This was further augmented by sampling a large variety of parameters, which ensures training samples have variable background noises, shade-off and halo amounts, and cell positions. This can be considered a form of data augmentation, but this step is performed before training the model, rather than during, and is based on the type of image one wants to segment. In total, each model is trained on 5000 unique synthetic images.

### Model training

We utilised two segmentation models in this work, DeLTA [30] (which is a U-net implementation) and Omnipose [31], an extension to Cellpose [32] allowing for the reconstruction of masks of arbitrary morphology.

The training protocol for each model is different. DeLTA was trained using pairs of synthetic images and ground truth masks for single colonies, but also required the calculation of weightmaps. These weightmaps are images with regions of high intensity corresponding to areas where masks are close to touching. During training, U-net pays additional attention to these regions to better learn segmentation of nearby, nearly touching objects. Therefore, if training DeLTA, SyMBac always generates binary masks which are not touching. Additionally, images and their masks need to be resized in order to fit within DeLTA’s architecture for training. This produces a model which accepts images of a certain dimension only. DeLTA was always trained with 4000 images of single trenches, for 400 epochs. We provide interactive notebooks to preprocess, train, and segment data with DeLTA.

Omnipose is trained differently. Omnipose can accept images of any size, and so we generated tiled images of 40 trenches at a time. This increased efficiency of training and allowed more total cells to be seen by the network. Additionally, when training Omnipose, masks are labelled, producing an instance segmentation. This allows masks to touch, which is beneficial in the segmentation of dense or closely packed bacteria. It is also beneficial in the segmentation of small cells, because no empty border needs to be created around each touching mask, which would be a significant fraction of the area of a small cell. Omnipose was always trained on 100 images of 40 trenches, for 4000 epochs. We provide notebooks for training and segmenting Omnipose based on the examples given from the original paper.

In both models’ cases, we opted to disable data augmentation, as we already sample mechanistic changes in the image formation process. Data augmentation can corrupt the image-object relation with unrealistic transformations. The only augmentations we allow are rotations and flipping of the data.

For mother machine experiments, we tested SyMBac against 4 datasets, which have their details given in Table 3. Three of the datasets are images of *E. coli* growing in a mother machine, taken with a 100× oil objective, and one dataset was imaged with a 40× air objective, using a 1.5× post-magnification lens, giving an effective magnification of 60×. Synthetic images for this dataset were still generated according to the optics of the 40× objective and then scaled to the appropriate pixel size.

**Table 3:** Datasets analysed using SyMBac

Dataset	Optics	Biology	Notable features
Lugagne et al. (exponential)	100× oil	*E. coli* in balanced growth	Poor contrast data
Bakshi et al. (exponential)	100× oil (NA = 1.30)	*E. coli* in balanced growth	High contrast data
Bakshi et al. (stationary)	100× oil (NA = 1.30)	*E. coli* in stationary phase	Small stacked cells
Bakshi et al. 60× (exponential)	40× air + 1.5× post mag (NA = 0.95)	*E. coli* in balanced growth	Low resolution + non-uniform illumination

Training data which contains a large variety of cell sizes can also be generated, with very small and very large cells being sampled. It is important to note that the model performance does not scale with the number of images of each cell type it sees, but rather by the raw cell count in each image. Therefore, if generating training data with a mixture of large and small cells, one should have fewer images of small cells, as they will contain more cells per image.

### Model evaluation

Because the training data is purely synthetic, validation data in the traditional sense does not exist. For this reason, during segmentation with either Omnipose or DeLTA, we saved the model after every epoch and evaluated performance through the analysis of growth data. Bacterial growth in the microfluidic linear colonies traces out a predictable sawtooth wave (Fig. S9.2 in Additional file 1). The first derivative of these sawtooth waves was analysed for spurious peaks, which are the signature of over- and under-segmentation errors (Fig. S9.3 in Additional file 1). The identification error rate is therefore the percentage of timepoints which contain a spurious peak. If a cell is mis-segmented between frames, it will appear as though a cell’s length sharply decreases then increases or sharply increases then decreases. These can be detected with a simple peak-finding algorithm, with a detailed discussion included in Additional file 1, Section 9.

### Segmentation analysis

The output from a U-net is a probabilistic threshold image. This image needs to be converted into a binary mask by thresholding pixel values between 0 and 1. Thresholds close to 0 will generate larger, connected masks, and thresholds close to 1 will generate smaller less connected masks. In order to identify the optimal threshold value which generates the most accurate masks, an independent set of synthetic data is segmented using the neural network, and the probability thresholds are adjusted to maximise the Jaccard index between the ground truth and the model output (Fig. S9.1b in Additional file 1). Depending on the type of dataset, this value can range between 0.6 and 0.99. If the thresholding value is low (around 0.6), then masks will be connected. To alleviate this, we use seeded watershed segmentation to cut masks appropriately. Seeds are generated by first thresholding masks at very high probabilities ($P>0.999$) and performing the watershed on the optimal probability binary mask. The output from Omnipose is an instance segmentation and requires no thresholding. It must be noted that we do not perform any post-processing on the training data or the segmentation results which would affect the shape of the mask.

In order to analyse segmentation precision, we looked at cell width and length. A single cell’s width is not expected to change drastically during growth. We tracked single cell width variation of models trained on Lugagne et al.’s human-annotated dataset, and synthetic data and plotted width distributions and time series to assess the difference in precision between the two models. This also gave a qualitative intuition of the temporal coherence of the models, as high variability will result in high temporal decoherence (Fig. S10.1 in Additional file 1). The cell length is expected to remain constant during the deep stationary phase. Using the dataset from Bakshi et al., we plotted each cell’s length during the stationary phase, and fitted a quadratic, taking the standard deviation of the residuals as our precision (Fig. S9.5 in Additional file 1).

After generation of the masks, information on cell curvature, radius (width), and length are calculated using the Colicoords Python library [16], with an example of the output coordinate system shown in Fig. 5c. While this analysis is slow (analysis of 1 million cells from the 100× Bakshi et al. dataset taking more than 48 h to complete on an AMD Ryzen 7 5800X with 16 CPU threads), the accuracy and performance of this type of analysis are unmatched when compared to the more commonly used functions for morphological analysis, such as fitting ellipses using regionprops from MATLAB or Scikit-image. The length and width of mothers can then be plotted over time from the output data.

## Supplementary Information

**Additional file 1.** PDF with additional information supplementing the main text, describing detailed implementation, usage, and analysis details: Section 1: Benchmarking Performance of Traditional Methods of Generating Cell Masks. Section 2: Details of Agent Based Model and Rigid Body Physics Simulation. Section 3: Details of OPL Calculation and Scene Drawing. Section 4: PSF Definitions, Convolution, and Image Simulation. Section 5: Comparison Between 3D and 2D PSF Models. Section 6: Implementation of the Camera Noise Model. Section 7: Image Optimisation. Section 8: Comparison of the Camera Noise Model with *ad hoc* Noise Matching. Section 9: Model Evaluations and Segmentation Precision. Section 10: Temporal Coherence of SyMBac Trained Models. Section 11: Segmentation Examples: Kymographs. Section 12: Size Regulation Analysis During Exit From Stationary Phase. Section 13: Extension to 2D Growth Regimes and Other Microfluidic Geometries.

## Data Availability

SyMBac’s documentation and API reference is available at https://symbac.readthedocs.io [44]. Datasets analysed are taken from [3] and [30], detailed in Table 3. Datasets are available at [45, 46]. $\bullet$ Project name: SyMBac $\bullet$ Project homepage: https://github.com/georgeoshardo/SyMBac [47] $\bullet$ Project documentation and datasets: https://symbac.readthedocs.io/en/latest/index.html [44] $\bullet$ Operating systems(s): Platform independent $\bullet$ Other requirements: Python 3.8 or higher $\bullet$ Licence: GPL-2.0

## Abbreviations

SyMBac: Synthetic Micrographs of Bacteria
ML: Machine learning
PSF: Point spread function
OPL: Optical path length
ABM: Agent-based model
API: Application Programming Interface
