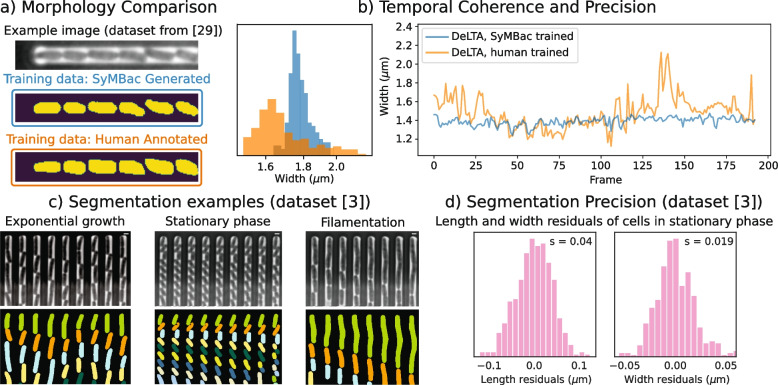# Correction: Synthetic Micrographs of Bacteria (SyMBac) allows accurate segmentation of bacterial cells using deep neural networks

**DOI:** 10.1186/s12915-025-02208-9

**Published:** 2025-04-16

**Authors:** Georgeos Hardo, Maximilian Noka, Somenath Bakshi

**Affiliations:** Department of Engineering, University of Cambridge, Trumpington Street, Cambridge, UK

**Correction** **:** **BMC Biology 20, 263 (2022)**

**https://doi.org/10.1186/s12915-022–01453- 6**

Upon publication of the original article [1], the authors noticed that Figure 4 contained an error.

The labels of the two traces presented in Fig. 4b are flipped in the figure legend, which causes the misinterpretation that DeLTA, SyMBac trained is noisier than DeLTA, human trained.

This conflicts with the histogram of Figure 4a, and the other relevant sections of the paper.

The correct color should be blue for DeLTA, SyMBac trained and orange for DeLTA, human trained.

The corrected figure can be viewed ahead in this Correction article.