# Supplementary material for: Synthetic Micrographs of Bacteria (SyMBac) allows accurate segmentation of bacterial cells using deep neural networks
Source: BMC Biol. 2022 Nov 30;20:263. doi: 10.1186/s12915-022-01453-6 (PMC9710168; doi:10.1186/s12915-022-01453-6)
Supplement: Supplementary file 1 — Additional file 1. PDF with additional information supplementing the main text, describing detailed implementation, usage, and analysis details: Section 1: Benchmarking Performance of Traditional Methods of Generating Cell Masks. Section 2: Details of Agent Based Model and Rigid Body Physics Simulation. Section 3: Details of OPL Calculation and Scene Drawing. Section 4: PSF Definitions, Convolution, and Image Simulation. Section 5: Comparison Between 3D and 2D PSF Models. Section 6: Implementation of the Camera Noise Model. Section 7: Image Optimisation. Section 8: Comparison of the Camera Noise Model with ad hoc Noise Matching. Section 9: Model Evaluations and Segmentation Precision. Section 10: Temporal Coherence of SyMBac Trained Models. Section 11: Segmentation Examples: Kymographs. Section 12: Size Regulation Analysis During Exit From Stationary Phase. Section 13: Extension to 2D Growth Regimes and Other Microfluidic Geometries. [file 12915_2022_1453_MOESM1_ESM.pdf]

Additional File 1 to: Synthetic Micrographs of Bacteria (SyMBac) Allows Accurate Segmentation of Bacterial Cells Using Deep Neural Networks

Georgeos Hardo[1], Maximilian Noka[1], and Somenath Bakshi[1,*]

[1]Department of Engineering, University of Cambridge, CB2 1PZ, UK
[*]Corresponding author: Somenath Bakshi, sb2330@cam.ac.uk

October 2022

Contents

1 Benchmarking Performances of Traditional Methods of Generating Cell Masks **2**

1.1 Human Drawn Masks 2

1.2 Membrane Dyes for Generating Cell Contours 3

1.3 Otsu's Method for Grayscale Thresholding 4

2 Details of Agent Based Model and Rigid Body Physics Simulation **4**

3 Details of OPL Calculation and Scene Drawing **7**

4 PSF Definitions, Convolution and Image Simulation **9**

5 Comparison between 3D and 2D PSF models **10**

6 Implementation of the camera noise model **12**

7 Image Optimisation **13**

8 Comparison of the camera noise model with *ad hoc* noise matching **15**

9 Model Evaluation and Segmentation Precision **15**

10 Temporal Coherence of SyMBac trained models **17**

11 Segmentation Examples: Kymographs **19**

11.1 Exponential growth (100x oil) 19

11.2 Entry to stationary phase (100x oil) 20

11.3 Exponential Growth (60x air) 21

12 Size regulation analysis during exit from stationary phase **21**

13 Extension to 2D growth regimes and other microfluidic geometries **22**

13.1 2D microfluidic growth chamber (microfluidic turbedostat) 22

13.2 Simulating growth of 2D colonies on agar pad 22

13.2.1 Typical Phase Contrast Image Features in Images of Monolayer Colonies on Agar-pad . . 22

13.2.2 Fluorescence Images of Monolayer Colonies on Agar Pad 25

1 Benchmarking Performances of Traditional Methods of Generating Cell Masks

To compare the accuracy of other segmentation methods, such as human annotated training data, traditional Otsu thresholding, and cell perimeter evaluation using membrane dyes, we generated synthetic images of single cells in phase contrast and with membrane dyes, along with accompanying ground truth. We then segmented the cells with Otsu's method and the membrane dye method, comparing the output masks to the ground truth. We show in this section that these methods systematically underestimate the cell's dimensions.

1.1 Human Drawn Masks

In order to test how accurate humans are at annotating images to generate training data, we sent 3 researchers a set of 100 synthetic images. The researchers were asked to both label the images and time themselves. The labelling was performed by sending each researcher a Python script which would open a napari window [1], and allow them to manually segment cells, saving their output to a file. We then compared the length, width, and pixelwise (IoU) accuracy of the human generated masks to the ground truth masks of the synthetic data. The IoU output of this result is given in Figure 1i, showing that humans consistently perform poorly in segmenting cells, especially if they are small and in stationary phase. We also observed that there was a significant bias in the way in which humans were mis-segmenting data, based on the cell's dimension and growth phase, shown below in Figure 1.1

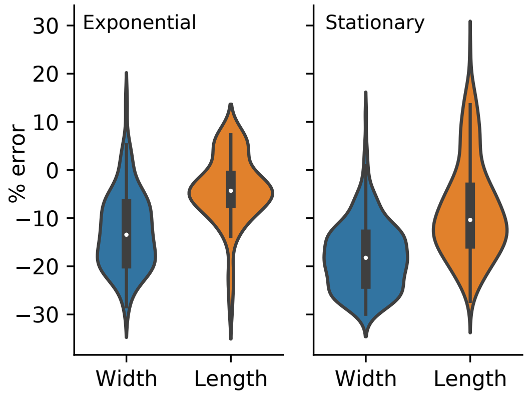

Figure S 1.1: The human annotation error is quantified by 3 researchers who were asked to manually segment 100 synthetic images. Their segmentations were compared against the ground truth. The worst performance was always in the width dimension with this becoming worse when the cells were in stationary phase. There was also a systematic tendency for human segmentations to underestimate the cell's dimensions on average.

1.2 Membrane Dyes for Generating Cell Contours

Next we show that using membrane dyes for estimation of cell size using diffraction limited imaging leads to severe underestimation of cell perimeter. We simulated images of cells tagged with a membrane dye by modelling cells as spherocylinders. We then assigned high fluorescence intensity values to the hull of the cell and performed 3D convolution of a fluorescence PSF approximated as a 3D Gaussian with appropriate X,Y and Z parameters. We then compared the true width of the cell with what one would infer if they assumed the cell width was the inter-peak distance of the maximal fluorescence intensities across the cell's minor axis. The simulations and a sample of a cell are shown below in Figure 1.2.

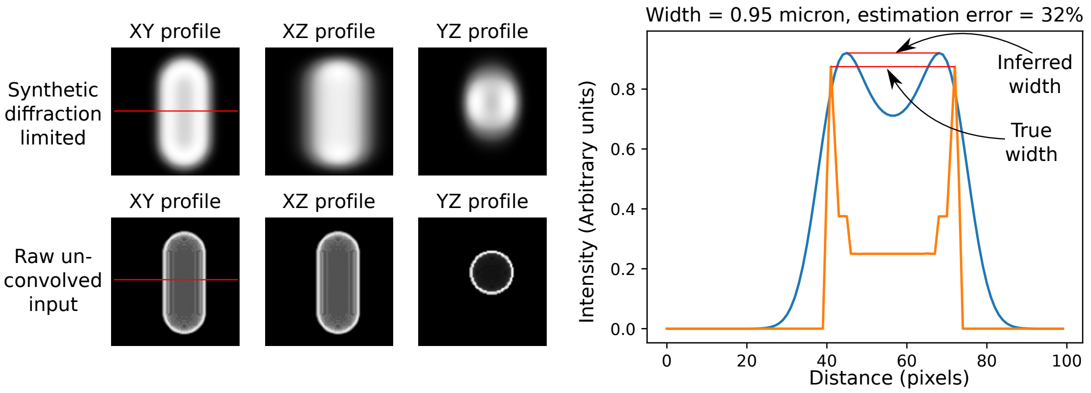

Figure S 1.2:
Left: Simulation of cells tagged with membrane dyes. Red line shows the slice taken for the intensity profile in the XY plane. Membrane dyed cells are simulated as hollow spherocylindrical hulls, with 0 intensity in the cytoplasm, and an intensity of 1 in the membrane. 3D convolution is then done with a Gaussian approximation to the fluorescence PSF to simulate the raw microscope image.
Right: Sample intensity trace of the intensity across the width of a cell. Blue shows the intensity of a diffraction limited image of a membrane dye tagged cell, and orange shows the true intensity profile. The microscope's optics corrupt the image and lead to a decrease in the inter-peak distance which subsequently underestimates the cell width.

This error becomes increasingly large as as the cell's width gets ever smaller. Figure 1.3 shows the error rates for width estimations for simulated cells from 0.6 microns to 1.4 microns in width. A very narrow cell, with a width of 0.6 microns would have a width estimation error of close to 80%. This represents a best case scenario, as the images were convolved with an ideal point spread function, and no noise was added to the image. In reality the estimation error will be worse.

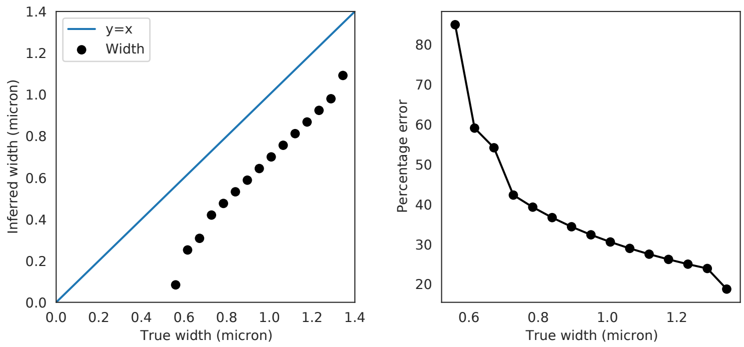

Figure S 1.3: Left: there is a systematic error in measuring the cell width using membrane dyes. Width is consistently underestimated. Right: The error in width estimation by using the inter-peak distance of membrane dye intensity is very high. A typical cell with a width of 1 micron will suffer an estimation error of more than 30%. Additionally for very thin cells it becomes impossible to make out the perimeter due to reaching the diffraction limit.

If membrane dyes are the only way to measure width due to experimental constraints (e.g the lack of a

phase contrast objective), then packages such as ColiCoords [2] are recommended, as they can mitigate the error incurred by this method by allowing the user to choose the method of perimeter estimation.

1.3 Otsu's Method for Grayscale Thresholding

While Otsu's method alone rarely yields good results for the segmentation of phase contrast cells, it is often used in combination with other preprocessing steps in the generation of training data for further use in machine learning pipelines. Thus its error must be investigated, because if it is used in the training data generation pipeline, its error will be propagated to the learning algorithm chosen. We simulated single cells imaged under phase contrast optics in order to quantify the error from this thresholding method compared to the ground truth mask. The error is systematic and constant across cell widths, meaning that narrower cells suffer from greater relative error rates in dimension estimation (Figure 1.4).

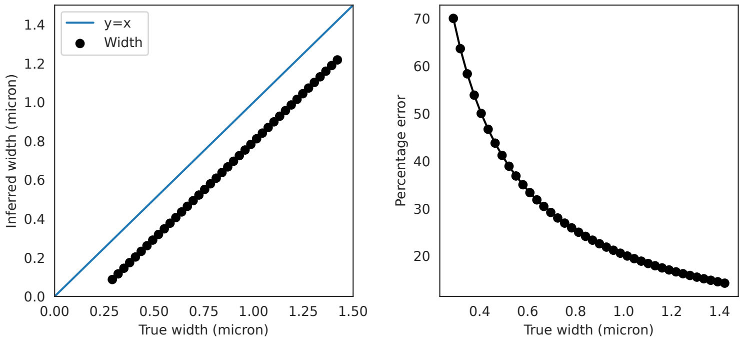

Figure S 1.4:
Left: The error from Otsu's method is a consistent offset for all cell sizes. While one could in theory compensate for this, the offset will not be immediately calculable as each microscope's PSF will slightly vary.
Right: the relative percentage error grows quickly as cells become narrow.

These data show that traditional methods of segmenting cells fail to accurately measure a cell's dimensions. Membrane dyes and Otsu's method does not account for the 2D projection effects and underestimates the cell width. These underestimation errors would be propagated to a learning based algorithm if used to generate training data. Further segmentation methods, such as those based around local thresholding and watershed could also suffer from errors in dimension estimation. This is because one needs to constantly tweak subjective parameters until the segmentation "looks right". For bioimage analysis of large objects, such as eukaryotic cells, this is typically not a problem, but as shown, it manifests for small objects are close to the diffraction limit, such as bacteria.

2 Details of Agent Based Model and Rigid Body Physics Simulation

Our cellular simulation is an agent based model, where each cell is defined by a number of attributes (`length`, `width`, `resolution`, `position`, `angle`, `growth_rate_constant`, `max length`, `max_length_var`, `width`, `width_var`). Cells can grow according to the adder, sizer or timer growth model.

For the spatial component of the simulation we used Python bindings for the popular Chipmunk physics engine, called Pymunk [3] in order to create a custom simulation environment. Cells exist as dynamic objects in a Pymunk space and can move around. Each time-step, the lengths of the cells are updated. This causes some cell hulls to overlap, the physics engine is then called to resolve these conflicts and move cells. While simulations most often produce realistic cell-stacking dynamics, the nature of cell-cell and cell-trench interactions can be varied by adjusting the number of physics iterations in each time-step, and by adding gravity to the simulation.

Stronger gravity in the direction of the bottom of the trench will induce tighter cell stacking, and in some cases double-loading of cells into the mother machine (which is a common occurance in experiments where the trench is too wide for the organism of choice). Low gravity, or negative gravity results in cells seemingly repelling each other, keeping a large distance from one another in the mother machine (often seen with motile strains which can leave trenches mid-experiment). Due to the unpredictability of cell stacking dynamics in real experiments (which vary due to media flow rate, cell size, trench dimensions, etc) we leave these parameters free to be changed by the user in order to maximise the similarity of the simulation to their experiment. The simulation can be watched in real time while it is running (Figure 2.1).

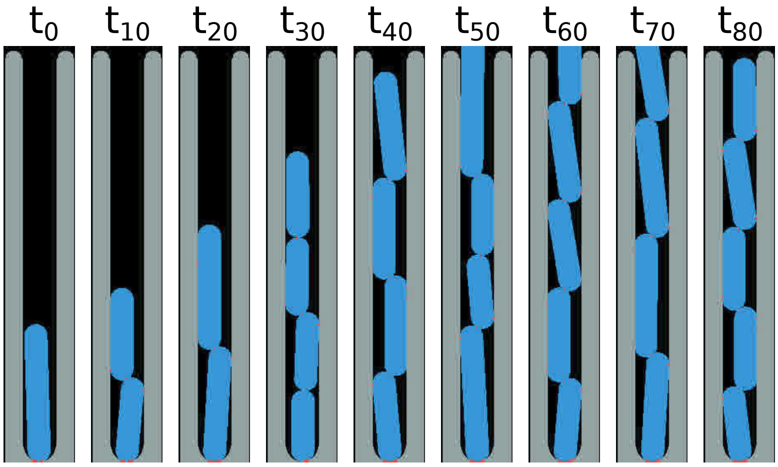

Figure S 2.1: Example kymograph of cells in the trenches from a simulation run. The user can monitor this simulation in real time and adjust parameters to achieve the desired result.

The simulation is defined by the user using the following parameters.

- `sim_length` is the number of simulation frames to run.
- `trench_length` is the length of the mother machine trench in microns.
- `trench_width` is the width of the mother machine trench in microns.
- `cell_max_length` is the mean maximum length of a cell in microns.
- `cell_width` is the mean cell width in microsn.
- `gravity` is a parameter which can be used to add or remove pressure of cells on one another. This is useful if for some reason cells in the simulation are falling into each other (although you should change `phys_iters` first if this is happening).
- `phys_iters` is the number of physics iterations per frame. 20 is a good starting point.
- `max_length_var` is the variance of the mean maximum cell length.
- `width_var` is the variance of the mean cell width
- `save_dir` is the directory to save the simulation output if it will be to analysed later, or with other code.
- `do_transformation` is a `bool` that decides whether cells are given a curve after the simulation, during rendering.
- `lysis_p` is the probability for a cell to lyse in each timepoint. Useful if generating training data for experiments involving antibiotics or bacteriophage.

The pseudocode below also demonstrates the broad overview of how the agent based model is run, treating cells as objects with properties which are modified every timestep.

Algorithm 1 Pseudocode for agent based simulation for bacterial growth

```
cell_length_var ~ N(max_cell_length, length_var)
cell_width_var ~ N(mean_cell_width, width_var)
angle_jitter ~ U(−0.01, 0.1)
lysis_p = 0.1
initialise first cell
add cell to population
for t in sim_length do
    for cell in cell_population do
        lysis ~ U(0, 1)
        if cell.y > trench_length or lysis < lysis_p then
            remove cell from population
        else if cell.length > cell.max_length then
            cell.dividing = True
        end if
        if cell.dividing then
            cell_length_var ~ N(max_cell_length, length_var)
            cell_width_var ~ N(mean_cell_width, width_var)
            septum_pos ~ N(0, cell.length/4)
            initialise daughter cell object
            daughter.max_length = max_cell_length + cell_length_var
            daughter.width = mean_cell_width + cell_width_var
            daughter.length = cell.length - septum_pos
            daughter.pos_x = cell.pos_x - septum_pos · sin(cell.angle · 2)
            daughter.pos_y = cell.pos_y - septum_pos · cos(cell.angle · 2)
            daughter.angle = cell.angle + angle_jitter
            add daughter to population
            cell.position_x = cell.position_x + septum_pos · sin(cell.angle · 2)
            cell.pos_y = cell.pos_y + septum_pos · cos(cell.angle · 2)
            cell.length = septum_pos
        else
            I ~ U(0, 7, 1.3)
            cell.length = cell.length·(1 + growth_rate · dt · I)
        end if
    end for
    for i in phys_iters do
        jitter cells
        apply gravity
        resolve collisions
    end for
end for
```

3 Details of OPL Calculation and Scene Drawing

At every time-point, each cell's location and dimensions are kept track of and recorded. This data is used to redraw the entire scene in 3D. While the simulation itself is not performed in 3D, with all interactions restricted to the XY plane, it is important to simulate the optics in 3D. The optical path length (OPL) of each pixel in the image must be simulated correctly. This is the optical distance that a ray of light experiences while travelling through the sample, and is given by a product of the geometric (real) distance travelled, and the refractive index of the medium which the light passes through. Our simulation has 3 main objects which light can pass through: the PDMS of the mother machine (referred to as the *device*), the cell growth medium, and the cell itself (Figure 3.1). This corresponds to 3 refractive indices. PDMS and growth medium have a constant depth in the XY plane, however the cells do not, this is the main reason for the importance of the 3D treatment of the cells. Light has a higher optical path length down the centre-line of a cell than at its edge.

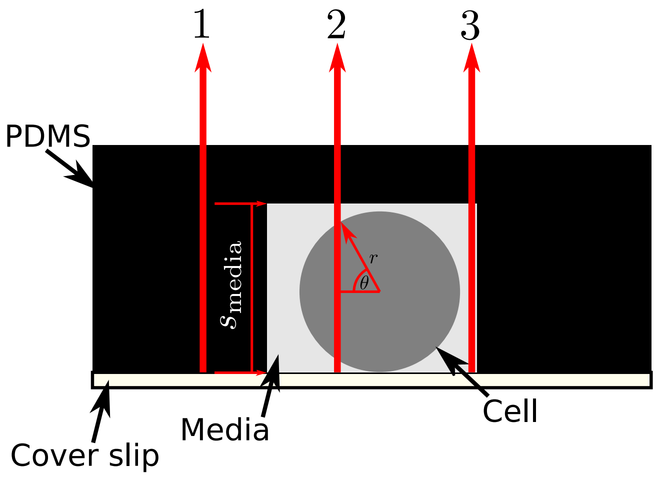

$$\text{OPL}_1 = n_{\text{PDMS}} \cdot s_{\text{PDMS}}$$
$$\text{OPL}_2 = n_{\text{cell}} \cdot 2 \cdot r_{\text{cell}} \cdot \sin(\theta) + n_{\text{media}} \cdot (s_{\text{media}} - 2 \cdot r_{\text{cell}} \cdot \sin(\theta)) + n_{\text{PDMS}} \cdot (s_{\text{PDMS}} - s_{\text{media}})$$
$$\text{OPL}_3 = n_{\text{PDMS}} \cdot (s_{\text{PDMS}} - s_{\text{media}}) + n_{\text{media}} \cdot s_{\text{media}}$$

Figure S 3.1: The OPL value is calculated for each pixel according to geometry and relative differences in refractive index, however the exact values of the refractive indices are left free to be optimised during the image generation step.

The 3D OPL images are then optionally modified to create curves in the cells. Each cell, being represented by an array, can be morphed to simulate cell curvature. In order to do this we roll individual rows of the cell image by a random number of pixels, with the transformation given by:

$$\text{roll}(\ell) = \begin{cases} 0 & \ell \leq 0 \\ a \sin(\frac{\ell \cdot \pi}{b} + c) & 0 \leq \ell \leq L \\ 0 & L \leq \ell \end{cases} \tag{1}$$

where L is the total length of the cell, l is the position down the length of the cell, and a, b, and c are randomly chosen for each transformation, but kept constant for individual cells. An example of the transformation of an OPL image viewed in the XY plane is shown in Figure 3.2

From the 3D OPL, masks are then generated. Two types of masks can be generated: instance masks, where each cell's mask is given a unique value, but no zero value pixels separate different cells, and semantic masks, where all cell masks have the same value (1), but cells are separated from one another with zero value pixels. Instance masks are intended for use with Star/Splinedist, while semantic masks are for use with DeLTA.

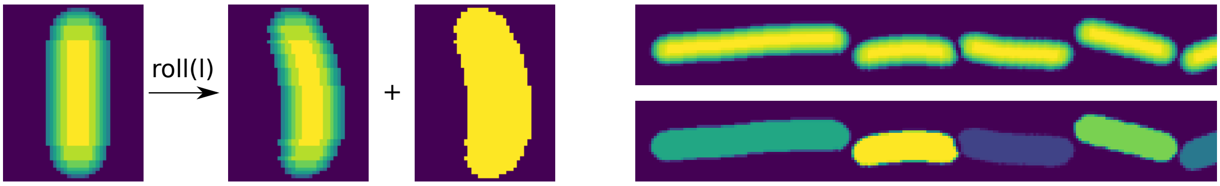

Figure S 3.2: Left: A random transformation of a straight cell (OPL image in XY) into a curved cell allows for rendering of more realistic images of mother machine images. The mask of the cell is then simply taken as the entire region defining the newly transformed cell. Right: Example output from the simulation of a single frame OPL image and its corresponding instance mask output.

After this process is completed, the trench is drawn around the cells according to the simulation input dimensions. Two new images, the image of the mother machine trench (trench image, $\boldsymbol{OPL_t}$), and the space in between the cells (media image, $\boldsymbol{OPL_m}$), are also derived from the simulation. The raw intensities of each image are then matched to the intensities of the trench, cells, and media in a real image. The user selects pixels in a real image corresponding to the cells, generating three arrays, $\boldsymbol{c}$, $\boldsymbol{t}$, $\boldsymbol{m}$, for cells, trench, and media respectively, with values of 1 where a pixel corresponds to that object, and 0 otherwise. The mean intensity is then calculated as:

$$\begin{aligned} I_c &= \frac{1}{MN} \sum_{m=0}^{M-1} \sum_{n=0}^{N-1} Y_{m,n} \cdot c_{m,n} \\ I_t &= \frac{1}{MN} \sum_{m=0}^{M-1} \sum_{n=0}^{N-1} Y_{m,n} \cdot t_{m,n} \\ I_m &= \frac{1}{MN} \sum_{m=0}^{M-1} \sum_{n=0}^{N-1} Y_{m,n} \cdot m_{m,n} \end{aligned} \tag{2}$$

Where M and N are the dimensions of the image, and $\boldsymbol{Y}$ is the image. The three OPL images are then summed as:

$$\mathbf{OPL} = \mathbf{OPL}_c \cdot I_c + \mathbf{OPL}_t \cdot I_t + \mathbf{OPL}_m \cdot I_m \tag{3}$$

We define a mask function, m as:

$$m(x) = \begin{cases} 0 & x = 0 \\ 1 & x \neq 0 \end{cases} \tag{4}$$

The image for the ground truth masks, $\mathbf{M}$, can then be defined as:

$$\mathrm{M}_{i,j} = m(\mathrm{OPL}_{c_{i,j}}) \tag{5}$$

4 PSF Definitions, Convolution and Image Simulation

After preparation of the OPL images, described in section 3, the PSF is prepared.

The fluorescence point spread function is modelled as a standard Airy disk, given by:

$$\mathrm{PSF}_{\mathrm{fluo}}(x, y) = \left[\frac{2J\left(2\pi \frac{\mathrm{NA}}{n\lambda} \cdot \sqrt{x^2 + y^2}\right)}{2\pi \frac{\mathrm{NA}}{n\lambda} \cdot \sqrt{x^2 + y^2}} \right]^2 \tag{6}$$

Where J is a Bessel function of the first kind, n is the refractive index of the imaging medium, NA is the numerical aperture of the objective, λ is the emission wavelength, and scale is the pixel-size.

The phase contrast PSF is modelled similarly to [4], as an obscured Airy disk given by

$$\mathrm{PSF}_{\mathrm{phase}}(r) = \left\{ \left[\frac{J(\omega)}{\omega} - (R - W)^2 \cdot \frac{J(2 \cdot (R - W)^2 \omega)}{\omega} \right] \right\} \tag{7}$$

with

$$\omega = 2 \cdot \frac{\pi \cdot \mathrm{NA}}{n\lambda} \cdot r \tag{8}$$

where R and W are the dimensions of the phase ring and condenser annulus (Figure 4.1). Example PSF images can be seen in Figure 4.2

The PSF is discretised by preparing matrix $\mathbf{R}$, of size K by L:

$$\mathrm{R}_{i,j} = \sqrt{(i - K/2)^2 + (j - L/2)^2)} \tag{9}$$

The discretised PSF matrix is thus:

$$\mathrm{PSF}_{i,j} = \mathrm{PSF}_{\mathrm{phase/fluo}}(r_{i,j}) \tag{10}$$

The phase contrast PSF is apodised using Gaussian apodisation, with the apodisation array being given by the function:

$$G(r, \sigma_1) = \exp\left(- \left(\frac{r^2}{2\sigma_1^2} \right) \right) \tag{11}$$

The apodised PSF is therefore given by

$$\mathbf{PSF}_{\mathrm{apodised}} = \mathbf{PSF} \odot G(\mathbf{R}, \sigma_1) \tag{12}$$

Where $\odot$ is the matrix elementwise (Hadamard) product. The theoretical minimum value for σ_1 can be thought of as being the microscope's diffraction limit, thus the phase contrast PSF cannot be smaller than a pure Airy disk. We use a Gaussian approximation for the Airy disk's [5] diameter for this estimate. Therefore we constrain σ_1:

$$\sigma_1 \gtrapprox 0.9\lambda \frac{n}{2NA} \tag{13}$$

Where λ is the imaging wavelength, n is the imaging refractive index, and NA is the numerical aperture of the objective. We then simulate defocus [6] in the image by convolving $\mathbf{PSF}_{\mathrm{apodised}}$ with another Gaussian:

$$\mathbf{PSF}_{\text{apo/def}} = \mathbf{PSF}_{\text{apodised}} * G(\mathbf{R}, \sigma_2) \tag{14}$$

where $*$ is the matrix convolution operation. Therefore, the first order, unoptimised version of the synthetic image can be created through the convolution:

$$\mathbf{S}_{\text{unopt}} = \mathbf{PSF}_{\text{apo/def}} * \mathbf{OPL} \tag{15}$$

All convolution takes place with edge reflection (using CuPy), however OPL images are intentionally drawn with large borders to avoid convolution edge effects. OPL images and kernels are rendered at at least 3x the imaging resolution. This ensures that when the convolution takes place, a high resolution kernel is convolved, and no details of the kernel's concentric rings are lost to pixelation. Only after convolution has taken place is the synthetic image then resized back to the camera's original pixel size.

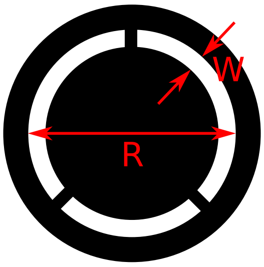

Figure S 4.1: Diagram showing the diemsions of the phase ring and condenser annulus to be used with Equation Equation 7 to parameterise the phase contrast point spread function.

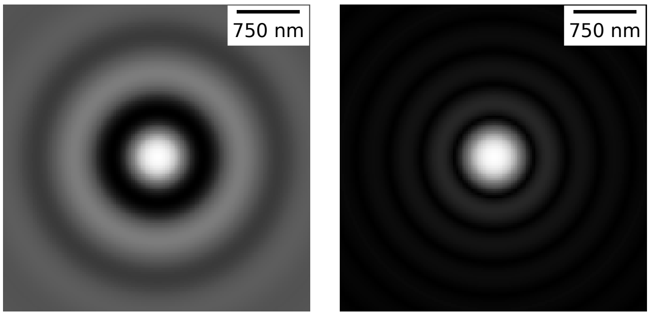

Figure S 4.2: Phase contrast (left) and fluorescence (right) kernels generated with $\lambda = 0.75$ micron, NA=0.97, $n = 1$, $W = 0.8$ mm, $R = 5$ mm. The phase contrast kernel is displayed with no apodisation or defocus. Both kernels have their intensities square rooted to enhance contrast for the reader.

5 Comparison between 3D and 2D PSF models

Taking the 2D projection of the OPL image, and convolving with an idealised 2D PSF is a simplification of the image generation process, trading off accuracy for performance gains in the convolution step. We sought to test whether a full 3D PSF model would have substantial effects on the quality of the training data, and thus on the performance of deep learning models. We generated synthetic data for 100x fluorescence images of bacteria in the microfluidic linear colonies. To do this, we reran the simulation step of the pipeline, but at the 2D projection stage of the pipeline, instead extracted 3D arrays of cell positions, where a 0 indicates no cell, and a 1 indicates the presence of cell material in that voxel. We generated 3D PSFs for each image using the model given by [7]. Convolution was performed on the OPL images with the 3D PSF, and the top down projection was taken by summing the resultant 3D synthetic image across its z axis. The same PSF parameters were then used to generate the equivalent 2D version by taking a projection. Convolution in 2D was then applied to the projection of the OPL image, generating a 2D synthetic image. The difference between both images was then

taken, and it was found that the maximal difference in pixel intensities was approximately 0.01% at its peak. It should be noted, that here, we refer to 3D convolution in a different manner than the traditional sense. Because 3D volumes are not being imaged but only projected in widefield microscopy (as the objective lens is kept at a fixed z position), we take 3D convolution to be the sum of 2D convolutions of each layer of the 3D PSF with the corresponding layer of the 3D OPL image. For instance, where a PSF and OPL image have Z layers, the resultant image, **S** is:

$$\mathbf{S} = \sum_{z=0}^{Z-1} \mathbf{PSF}_z * \mathbf{OPL}_z \tag{16}$$

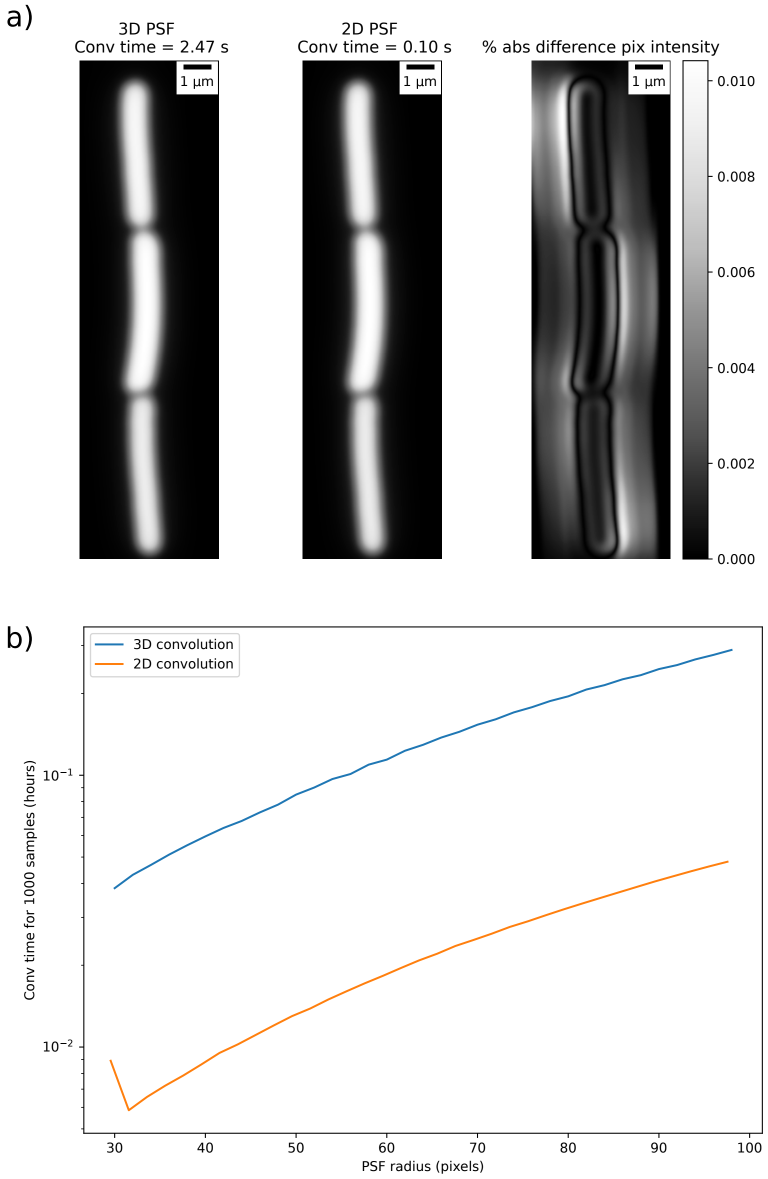

Figure S 5.1: Comparison between 2D and 3D fluorescence PSF convolution. The PSF models were generated according to [7]. **a)** shows the raw output of a 3D and 2D convolutions, with the corresponding time taken for the operation on an Nvidia Quadro RTX 4000 GPU. Also the percentage absolute difference in pixel intensities between each image can be seen. **b)** shows how the convolution time with the PSF changes as a function of its radius. Higher radius PSFs will simulate longer range diffraction effects, resulting in higher accuracy at the cost of performance.

Convolution of a 3D PSF is more than an order of magnitude slower than 2D convolution, and the difference in run times will grow if the synthetic images are rendered at higher resolutions. However, as the difference in the image output is minimal, we expect the performances of the segmentation networks trained with 2D or 3D

PSF models.

To test this, we generated two synthetic datasets of 500 images each which were identical except for the convolution mode. To test the effects of 3D vs 2D PSF convolution, we used this trianing data to train Omnipose and DeLTA to segment images of *B. subtilis* exiting stationary phase (100x Plan Apo oil objective, fluorescence and phase contrast channels). We found no significant difference in the identification accuracy of Omnipose (trained for 4000 epochs) and DeLTA (trained for 400 epochs) models trained with either 3D convolved data or 2D convolved data, nor did we find any significant difference in the mean lengths and widths of cells throughout the experiment (0.4% identification error rate with camera noise model). This implies that such small changes in the training data intensity do not have an effect on the quality of the model.

6 Implementation of the camera noise model

SyMBac allows for a simulation of the camera noise in the image formation process, instead of *ad hoc* noise matching, if the camera parameters are available to the user. We implemented the camera noise model described in the linear version of the EMVA1288 4.0 Standard for Measurement and Presentation of Specifications for Machine Vision Sensors and Cameras. EMVA1288-linear describes a pixel sensor which integrates a number of photons over time, filling a well of electrons, whose charge is converted to a voltage which is amplified and digitised into a greyscale signal. We consider the two main sources of noise; photon noise and dark noise, modelled as Poisson and Gaussian distributions respectively. The camera's sensitivity, dark noise, and baseline intensity are required to be known. If the dark noise cannot be found, or the camera's precise mode of operation is not known (which often affects the dark noise levels), then it can be estimated by capturing dark photos and calculating the standard deviation of the pixel intensities. Additionally with this method the baseline intensity can be calculated.

The advantage of a forward simulation of camera noise, is that it removes the need to use intensity, histogram, and noise matching when generating synthetic fluorescence data. Matching these properties of a synthetic image of cells in one orientation, to a real image of cells in another orientation is never ideal, and this can slightly corrupt cell widths.

The camera noise is added to the image after PSF convolution, described in section 4. We assume that the sensor is linear in ADUs vs photon count, and that each pixel has identical dark noise. We model the image shot noise (each pixel's charge unit variability) as a Poisson distributed random variable, with mean and variance equal to the number of accumulated electrons in each pixel. The sensitivity (s) of the sensor (in ADU/e^-) is therefore used to convert the image first into electrons. Therefore the matrix $\boldsymbol{A}$ is the shot noise in the image. The matrix $\boldsymbol{B}$ is the dark noise in the image. Each element is a random variable with mean equal to the baseline value of each pixel, and standard deviation equal to the camera's dark noise, both of which are parameters which can be obtained from most camera specifications.

$$\begin{aligned} A_{i,j} &\sim \text{Pois}(S_{\text{unopt}_{i,j}}/s) \\ B_{i,j} &\sim \mathcal{N}(\mu_d, \sigma_d^2) \\ S_{\text{noise}_{i,j}} &= s \cdot (A_{i,j} + B_{i,j}) \end{aligned} \tag{17}$$

In some cases, the values for μ_d and σ_d^2 are not available, or the camera's mode of operation is not known for a particular dataset. These values can, however, be calculated from taking K $M \times N$ dark images, $\boldsymbol{D}$ with the camera:

$$\mu_d = \frac{1}{sKMN} \sum_{k=0}^{K-1} \sum_{m=0}^{M-1} \sum_{n=0}^{N-1} D_{k,m,n} \tag{18}$$

If we assume that the temporal noise is stationary and homogeneous, then we can get a good estimate for σ_d^2 from a single image.

$$\sigma_d^2 = \frac{1}{sMN} \sum_{m=0}^{M} \sum_{N=0}^{N} (D_{0,m,n} - \mu_d)^2 \tag{19}$$

$\boldsymbol{S}_{\text{noise}}$ is now a synthetic image with matched intensities and noise, and is a good approximation of a real image. In some cases, however, further small modifications can be made to the image to increase similarity even further. These include matching the rotational Fourier spectra of the real and synthetic images, as well as matching the intensity histograms of the two images. The fourier matching algorithm is adapted from the MATLAB SHINE toolbox, which we have rewritten and optimised for Python [8], with out implementation detailed within the function documentation. Histogram matching uses the scikit-image implementation [9].

Estimating σ_1, the apodisation, and σ_2, the defocus, is difficult. Therefore these two parameters are left floating, interactively adjustable

7 Image Optimisation

After convolution and resizing, optional image optimisation takes place. The first optimisations are fine-tuning of the refractive index of the PDMS, media, and the cell. In order to get the correct intensity values for these features, the users are presented with an interactive Napari [1] window (Figure 7.1). The user must label (with any value > 0) individual layers corresponding to the cell, media, and the device. Accuracy is not needed as only simple estimate of the mean and variance of each intensity is required.

Next, the lens apodisation (modelled as the PSF multiplied by a 2D gaussian) and defocus (modelled as the PSF convolved with a 2D gaussian) can be specified by varying the sigma values in Equation 17. These optimisations alone are often sufficient to produce a highly similar image, however similarity can be maximised by the addition of noise and matching various properties to real images. Noise is modelled in two ways: either using the camera model described in section 6, or through the addition of Gaussian noise with user-controlled variance, and post-noise addition matching of intensity histograms with the real image. Histogram matching can be used even if the camera model has been used, as it resolves any remaining discrepancies in image intensity. Additionally, in some instances rotational Fourier spectrum matching can be beneficial, and this is implemented through a python translation translation of the SHINE toolbox [8]. Fourier spectrum matching is only recommended for high resolution and high magnification images, as its main purpose is to replicate the intricate texture found on cells (which is typically not visible on lower magnification images). In our pipeline we only turn on this setting for 100x oil images. All of these parameters can be adjusted interactively in an IPython notebook using sliders, shown below in Figure 7.1. The parameters are used to minimise an objective function:

$$\begin{aligned}\text{minimise} \Bigg\{ & \frac{\left| I_c - \frac{1}{MN} \sum_{m=1}^{m-1} \sum_{n=1}^{N-1} \cdot S_{\text{noise}_{i,j}} \cdot m(OPL_{c_{i,j}}) \right|}{I_c} \\ + & \frac{\left| I_t - \frac{1}{MN} \sum_{m=1}^{m-1} \sum_{n=1}^{N-1} \cdot S_{\text{noise}_{i,j}} \cdot m(OPL_{t_{i,j}}) \right|}{I_t} \\ + & \frac{\left| I_m - \frac{1}{MN} \sum_{m=1}^{m-1} \sum_{n=1}^{N-1} \cdot S_{\text{noise}_{i,j}} \cdot m(OPL_{m_{i,j}}) \right|}{I_m} \Bigg\}\end{aligned} \tag{20}$$

Or, simply, the objective function is minimising the sum of the three relative errors between the empirical cell, trench, and media intensities, and those in the final synthetic image, defined as e_c, e_t, and e_m.

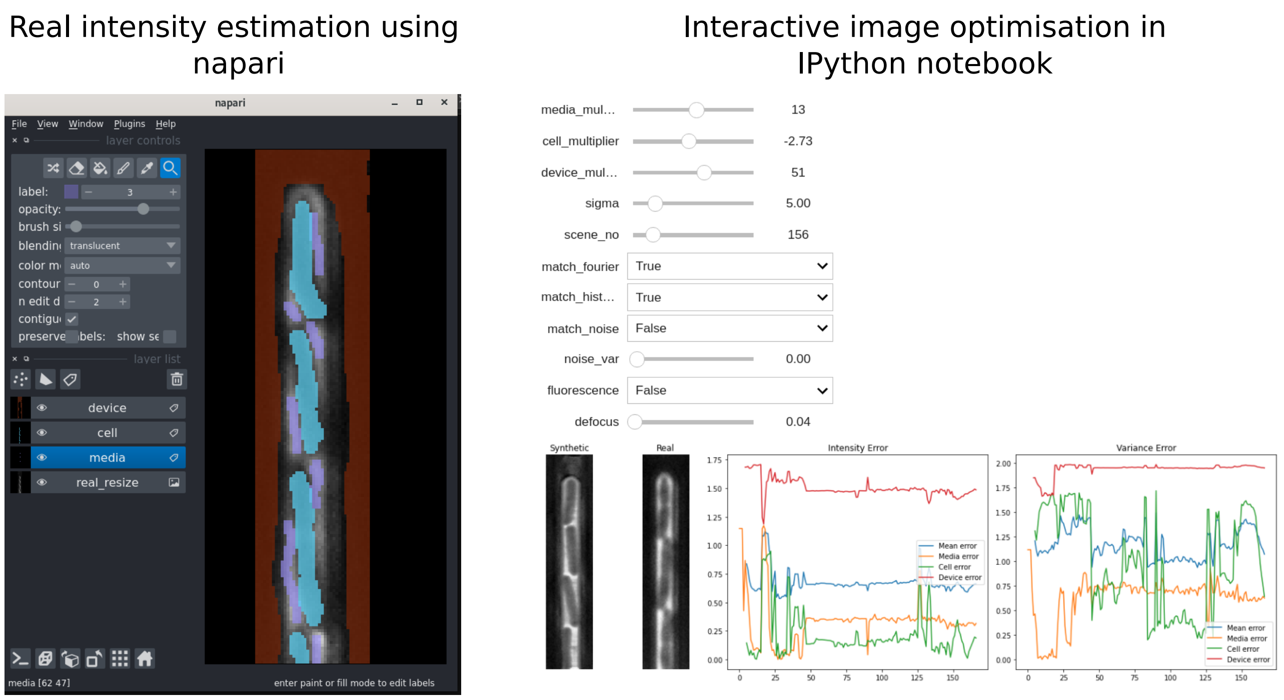

Figure S 7.1: Left: Labelling of a sample real image to identify image regions (cell, device, media) in order to adjust the intensities of the conrresponding regions in the synthetic image. Right: Example of the interactive adjustment interface mid-optimisation for a 40x image. Adjustment sliders for the media, cell and device intensities, apodisation sigma, noise and defocus values are available. Options are also available to toggle Fourier, histogram and noise matching as well as convenient switching to an equivalent fluorescence kernel.

The interactive notebook pictured is available as an example, along with sample images at `https://github.com/georgeoshardo/SyMBac/blob/main/examples/Drawing_Phase_Contrast_100x_oil.ipynb`. As per the documentation, the adjustable parameters are given by:

- `media_multiplier` is the intensity multiplier for the media part of the image.
- `cell_multiplier` is the intensity multiplier for cell parts of the image.
- `device_multiplier` is the intensity multiplier for the device part of the image.
- `sigma` is the radius (in pixels) of the gaussian apodisation of the phase contrast PSF (if you are using phase contrast).
- `scene_no` is the index for the frame of the synthetic images you rendered.
- `match_fourier` controls whether you are matching the rotational Fourier spectrum of the synthetic image to the real image.
- `match_histogram` controls whether you are matching the intensity histogram of the images with each other.
- `match_noise` controls whether you are matching the camera noise of the images with each other.
- `noise_var` controls the variance of the shot noise added to the image.
- `fluorescence` controls whether you are rendering a fluorescence of phase contrast image.
- `fluo_3D` Switch to 3D convolution with fluorescence PSF
- `camera_noise` Switch on camera noise simulation (if camera parameters have been supplied)
- `defocus` controls the radius of a gaussian which simulates depth of focus and out of focus effects of the PSF.

8 Comparison of the camera noise model with *ad hoc* noise matching

We generated two identical synthetic datasets of bacteria *B. subtilis* exiting stationary phase (100x Plan Apo oil objective) in both fluorescence and phase contrast, and trained Omnipose models on each of the 4 datasets (fluo+camera noise, phase+camera noise, fluo+noise match, phase+noise match) to segment the experiment. We found no difference in identification error (1.0%) or cell morphology when comparing phase contrast training data with camera noise, and with noise matching. This is likely due to the relatively high intensity values in phase contrast images, diminishing the effects of noise.

In fluorescence however, the effects of noise are large at these low intensities. Dark noise and even photon noise can make up a significant fraction of the image's intensity, lowering the signal to noise ratio. We found a significant difference in the identification error between models trained on fluo+camera noise data and fluo+noise match data. Models trained on the former had a 0.4% cell identification error rate, whereas models trained on the latter noise matched data had a 1.9% error rate when compared across single mother cells. The simulation of camera noise for fluorescent synthetic images has a significantly positive effect on the segmentation error rate. Interestingly, the difference in morphology of cells segmented with models trained on fluo+camera noise data and fluo+noise match data was not significantly different. Length and width distributions were not significantly different, and comparing masks across results gave a mean IoU of 0.83 ± 0.02.

9 Model Evaluation and Segmentation Precision

Figure 9.1 shows the error's decrease as training progresses, but the model can overfit. Therefore automated analysis of the segmentation errors (details in subsequent figures) is used to find the epoch which minimises the error.

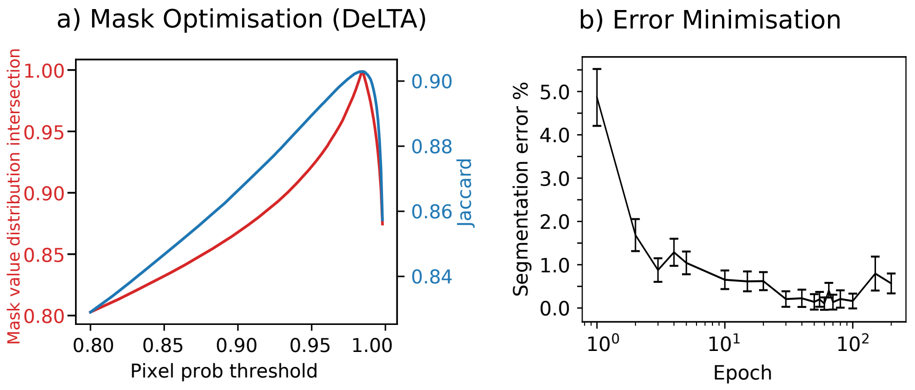

Figure S 9.1: a) The optimal probability threshold is identified by passing synthetic validation data through the model, and the threshold is adjusted to maximise the Jaccard index between the predicted masks and the ground truth masks, or the maximal distribution intersection between size distributions.
b) Segmentation error of trained models are evaluated automatically epoch-by-epoch by analysing the sawtooth curves of cell length changes over time, and the model with the lowest error is kept.

The growth profile of the individual cell lends itself well to automated detection of segmentation errors. The log transform of the length vs time traces out a saw-tooth wave with variable amplitude and phase. By taking the numerical derivative of this data one can find over and under-segmentation errors by simply searching for peaks over a certain threshold and with a certain prominence. These peaks can then be mapped back to the original length trace and the errors corrected with a variety of signal processing techniques.

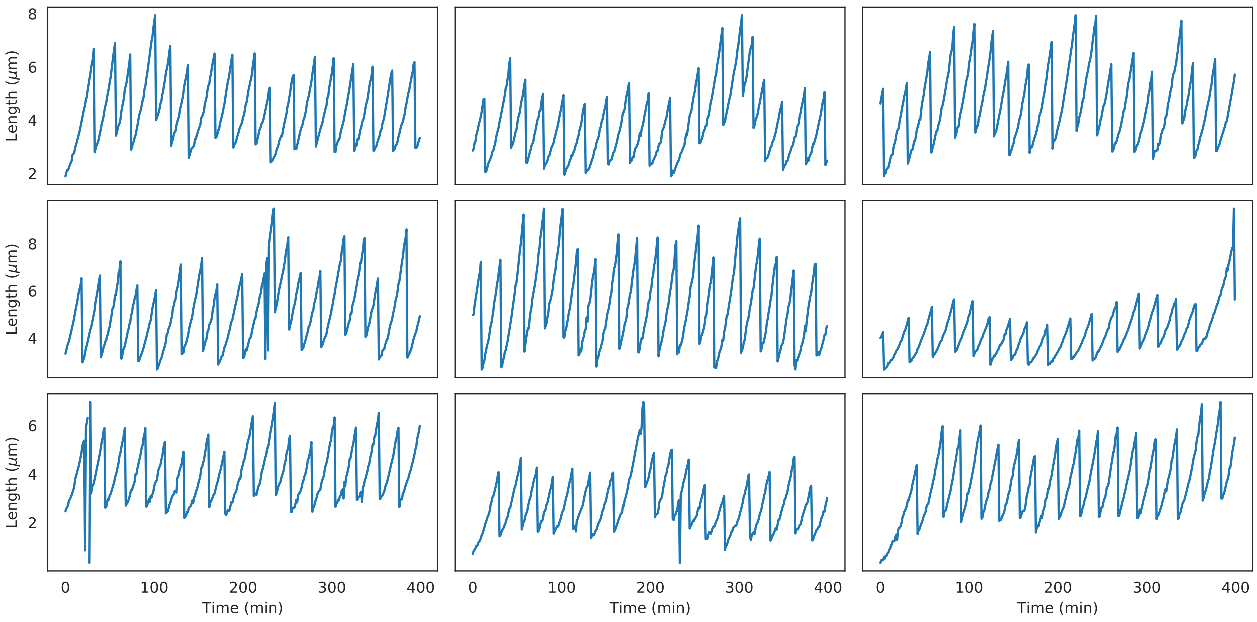

Figure S 9.2: A sample of cell length vs time plots, reminiscent of saw-tooth waves. (Segmented from 100x oil data, kymographs in Figure 11.1 and Figure 11.2

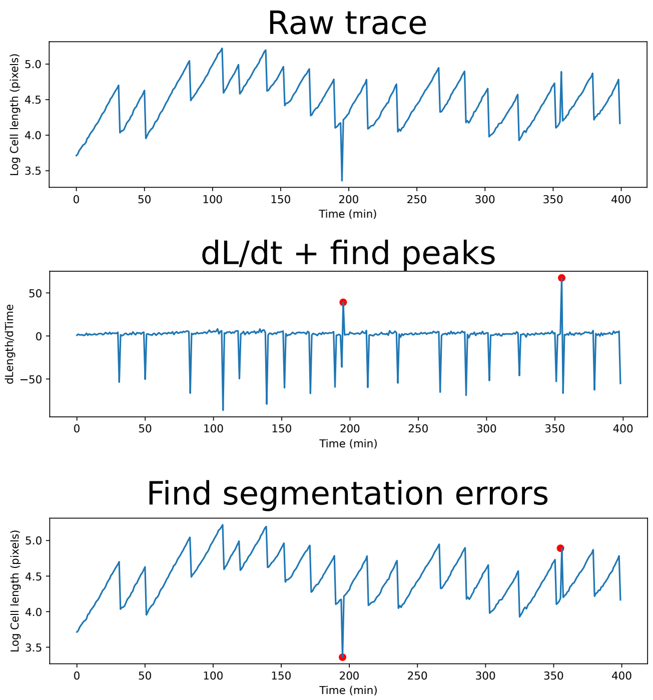

Figure S 9.3: Errors in the segmentation output's raw length trace are identified by taking its numerical derivative. Peaks in the numerical derivative are found and mapped back to the original data. In this case the errors are fixed simply by smoothing out peaks by setting them to the midpoint value of the adjacent values. For errors which span more than a single frame, errors can still be identified in this way, but require more sophisticated correction methods (for example rolling back by more than one frame, rolling forward by more than one frame, and interpolating between the frames).

Full traces reveal good temporal coherence, and the ability to accurately resolve width over time.

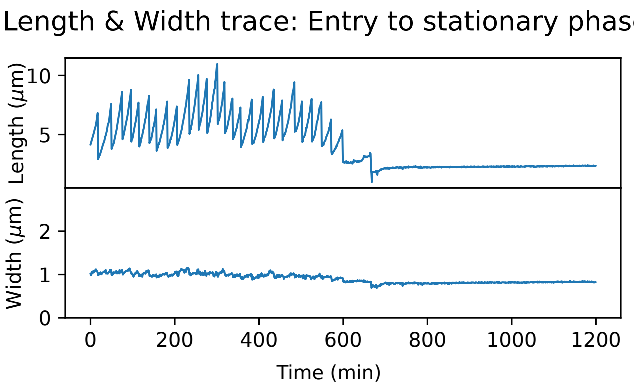

Figure S 9.4: An example cell length vs time trace of a cell entering stationary phase. As can be seen from the width and length plots during the stationary phase, there is very high temporal coherence, and the width can be studied at the sub-micron level. At stationary phase, the variance in the width was 100nm^2, and thus a precision of 10nm can be reached in the estimation of the cell width.

We show that we can achieve a precision of as low as 6.8 nm in the estimation of a cell's width, and a precision of 20 nm in the estimation of a cell's length in stationary phase (Figure 9.5). This is only possible due to the high quality training data fed to the model. This is proven by the comparison between human-made and synthetic training in Figure 2g.

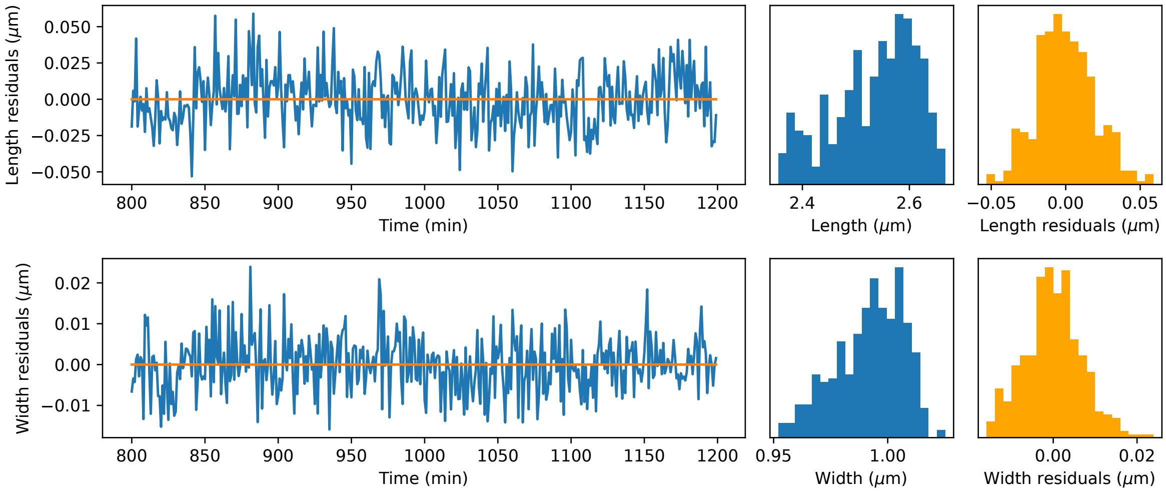

Figure S 9.5: In order to estimate the precision of width and length estimations in the stationary phase, we fit a quadratic polynomial to cell lengths and widths in stationary phase over time. As an example, we show the histograms of the true lengths and widths of this cell, along with histograms of the fit residuals. The standard deviation in the width residuals was 6.8 nm and the standard deviation in the length residuals was 20 nm.

10 Temporal Coherence of SyMBac trained models

In order to evaluate the quality of masks generated by models trained on human generated data and computer generated data, we compared the masks outputted by the pretrained model supplied with the DeLTA paper on its own test data, and a model we trained on synthetic data. We first made qualitative observations of mask quality, and then quantified these by assessing the temporal coherence of single cell width between frames. The results of this comparison can be seen in the histogram in Figure 2d, whereby the distribution of output mask widths is tighter for SyMBac trained models. This is further exemplified by noting that the temporal coherence of mask widths was poor as shown below. An example trace of cell widths is shown as a comparison of the outputs from the two types of training data in Figure 2d.

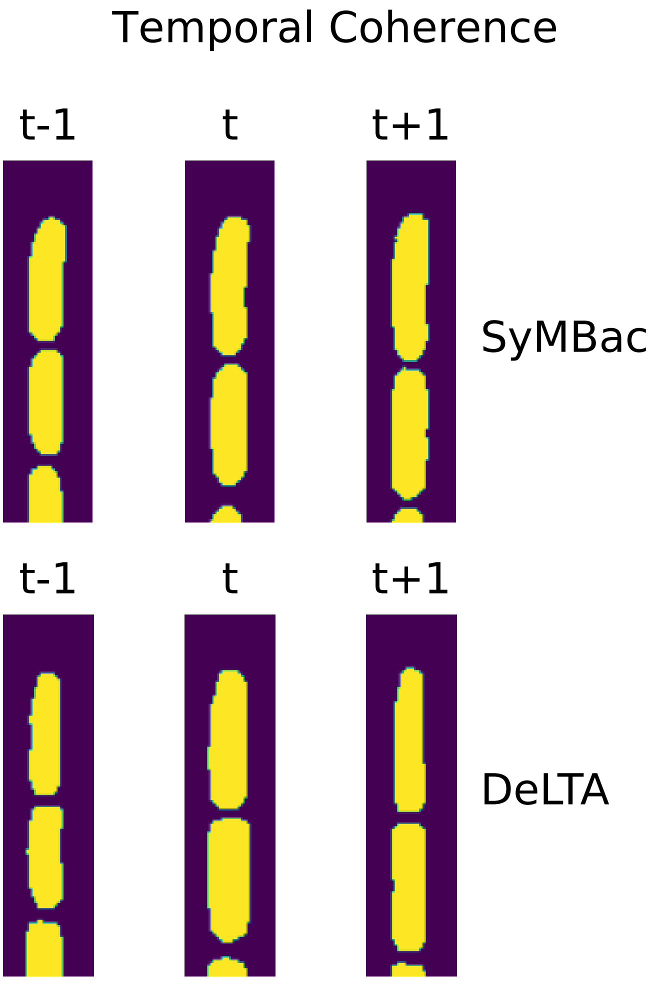

Figure S 10.1: Masks generated from models with SyMBac data can be seen to produce tighter mask width distributions, the result of which is reduced artefactual fluctuations in mask width, leading to higher temporal coherence. The output masks of models trained on the DeLTA training data show large and visible fluctuations in width from frame to frame, as exemplified.

11 Segmentation Examples: Kymographs

11.1 Exponential growth (100x oil)

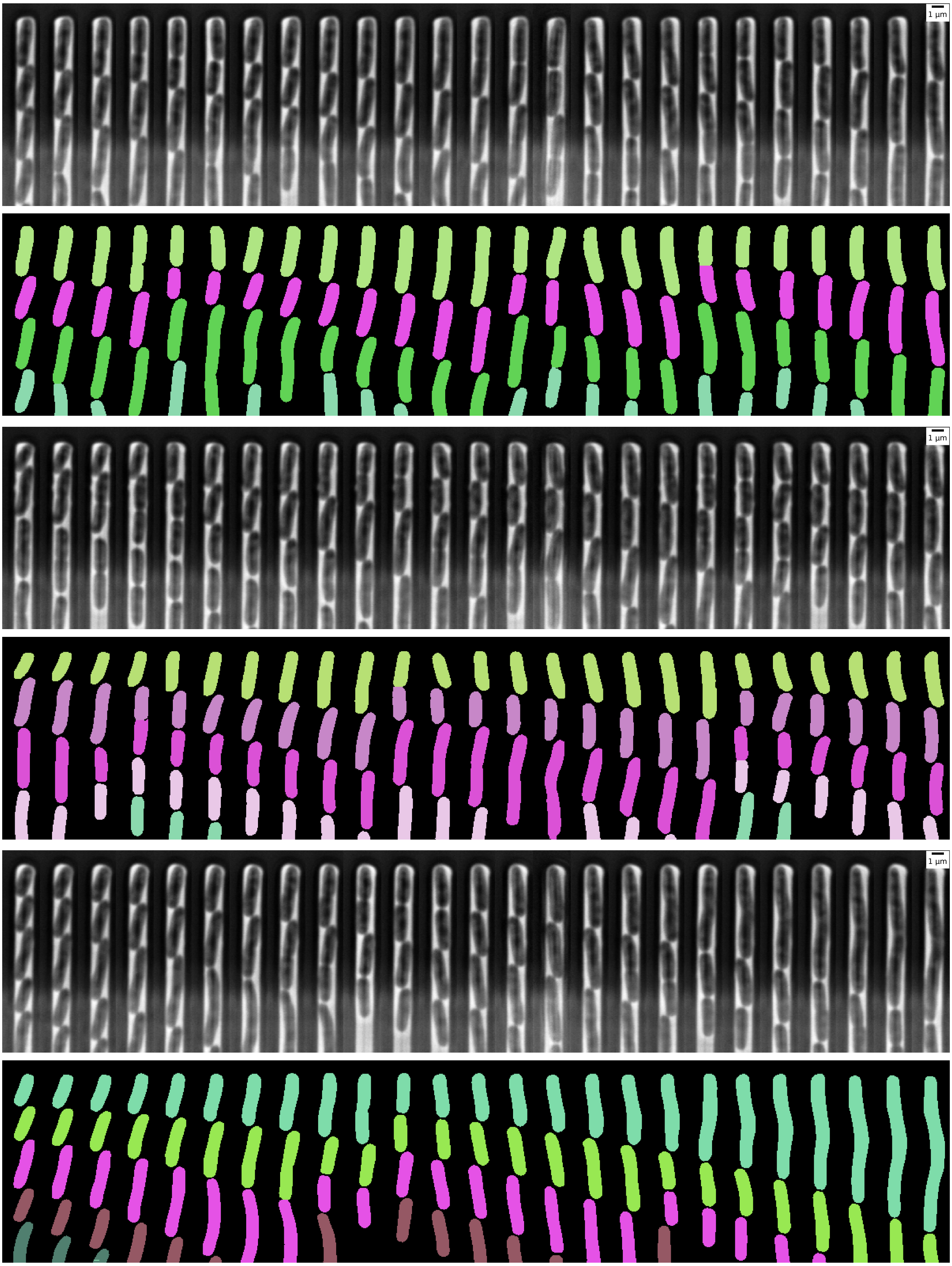

Figure S 11.1: Exponential growth in the mother machine, from Bakshi et al. [10]. Frame spacing is 3 minutes

11.2 Entry to stationary phase (100x oil)

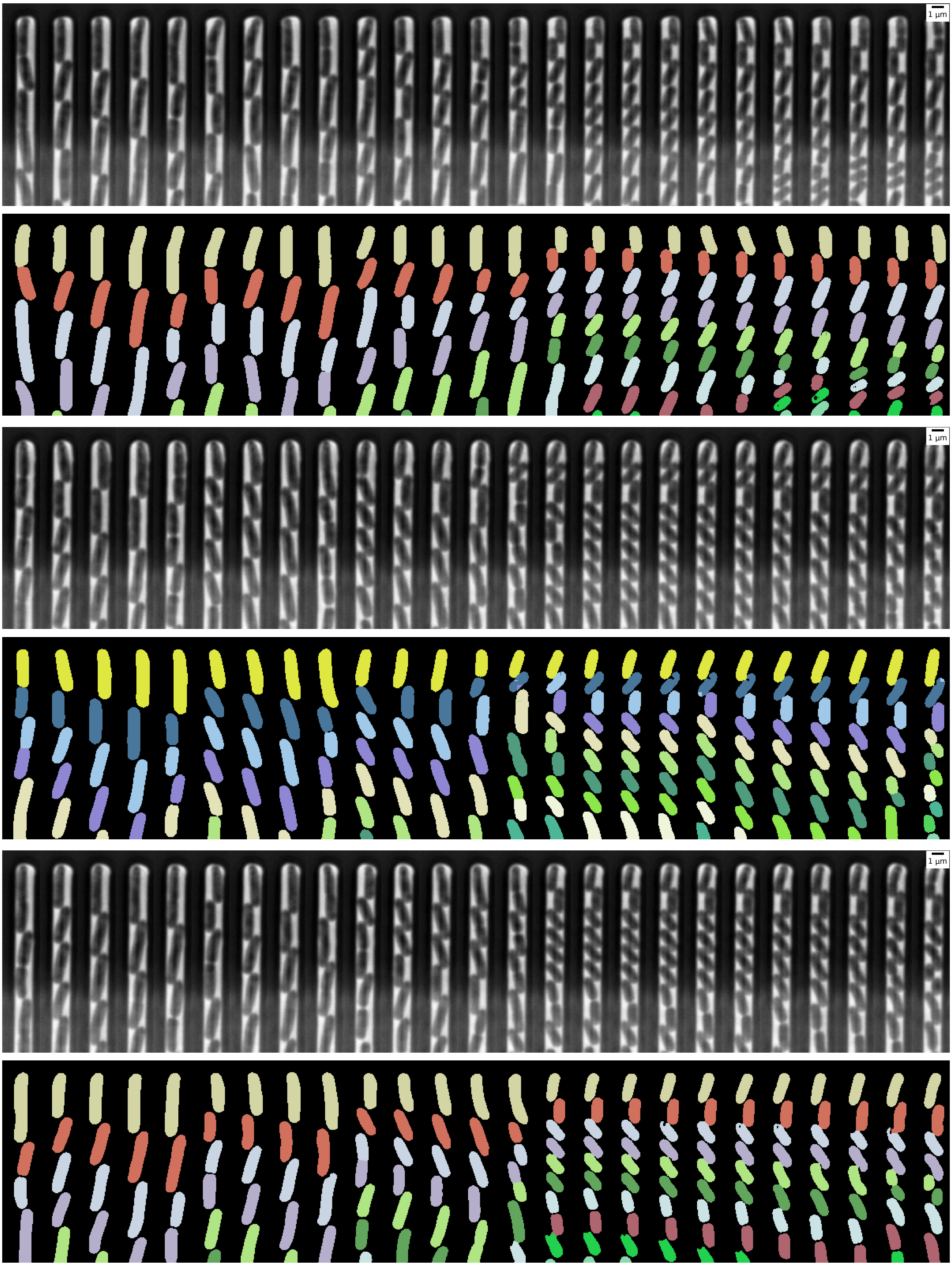

Figure S 11.2: Entry to stationary phase growth, from Bakshi et al. [10]. Frame spacing is 6 minutes

11.3 Exponential Growth (60x air)

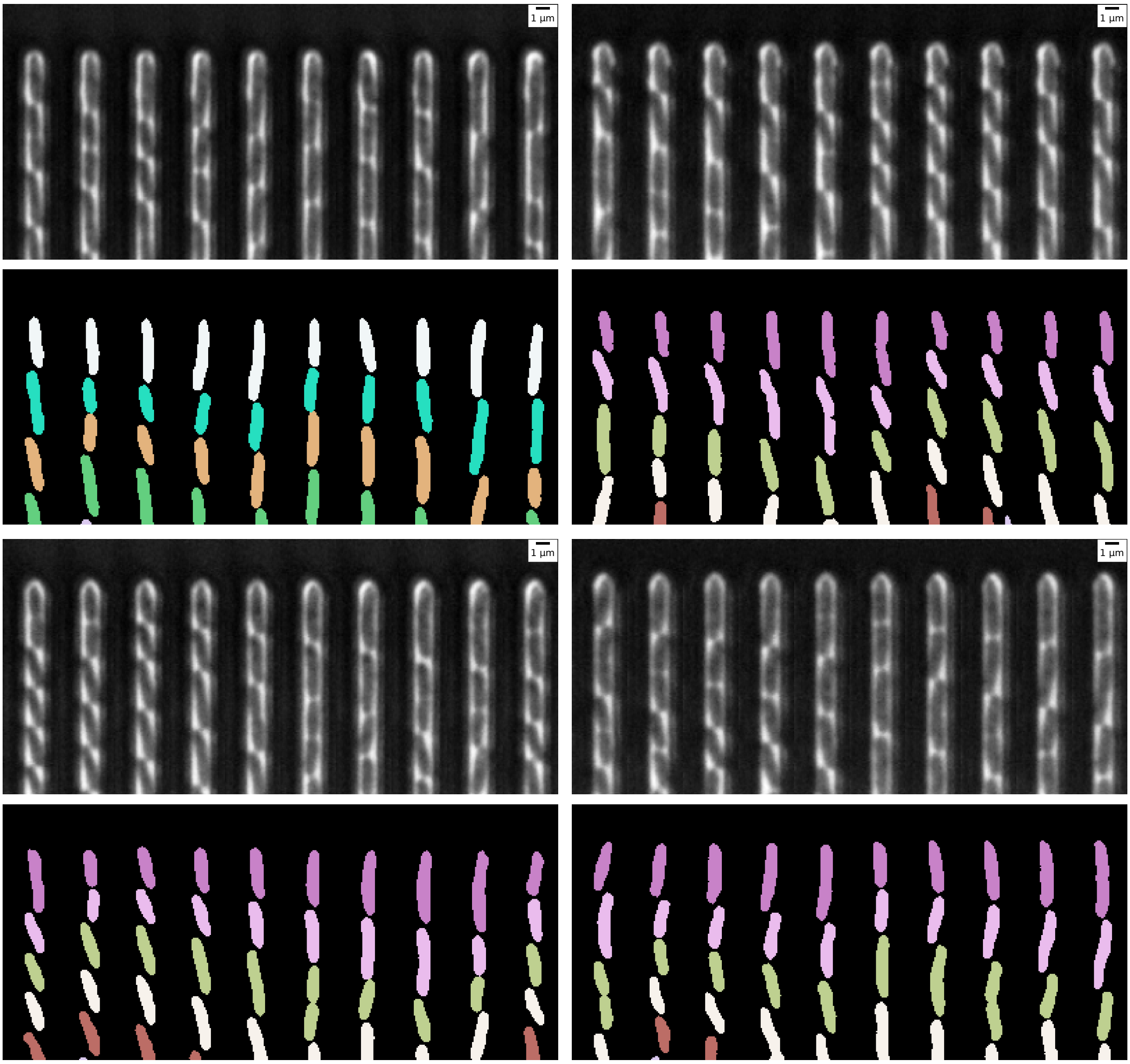

Figure S 11.3: Exponential growth in the mother machine, from Bakshi et al. [10]. Frame spacing is 5 minutes

12 Size regulation analysis during exit from stationary phase

Correlations between sizes (area), length, and width at the point of exit from stationary phase and at the first division indicates cells are sizers. Irrespective of the initial size, length, width, cells only divide after reaching a critical value.

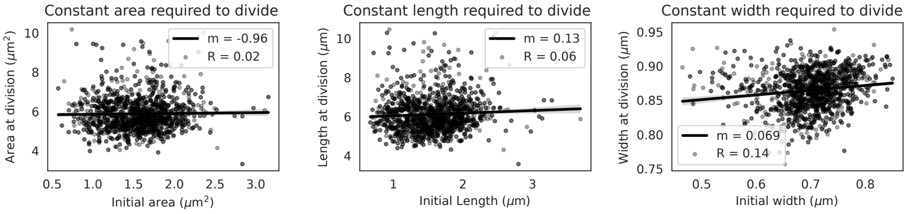

Figure S 12.1: The cells are sizers in every aspect, area, width and length, during exit from stationary phase.

13 Extension to 2D growth regimes and other microfluidic geometries

SyMBac works in much the same way for 2D growth as it does for 1D mother machine growth, with a few key differences. Firstly, the cell simulator back-end was switched from our custom simulator (built only with 1D growth in mind) to the more general CellModeller [11] (the reason for not using CellModeller for all simulations, is that CellModeller cannot vary cell width during a simulation, whereas our simulator can, allowing us to capture a variety of widths in the 1D growth data, where it matters most). Simulations of 2D growth (standard agar pad experiments) and channelled 2D growth like that seen using the microfluidic device in [12] were generated, and the cell properties saved. SyMBac was then used to redraw the scenes and apply filters to produce the synthetic data.

13.1 2D microfluidic growth chamber (microfluidic turbedostat)

Microfluidic devices need not be restricted to 1 dimensional growth. Increasing the width of a mother machine trench turns it into a microfluidic chemostat. Simulating cells in this geometry consists of simply adding two constraints to side of the simulation much in the same way as mother machine growth is simulated. Cells are then removed when they reach the horizontal nutrient flow. Due to the large halo often seen near the flow channel making cells difficult to see, we crop the image to only include the main region of growth. The generated data is then fed into a segmentation network, and an example montage of the output masks is shown in Figure 13.1.

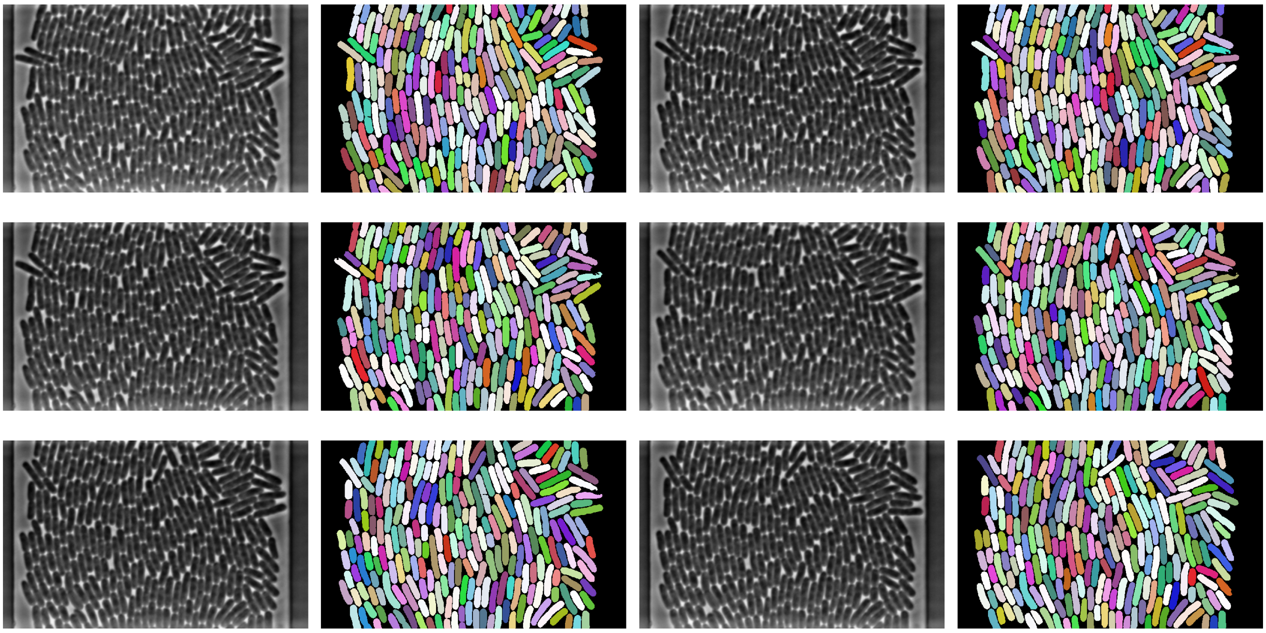

Figure S 13.1: Montage of segmentation outputs from a model trained on SyMBac data, from various sequential timepoints of data supplied by the Elf Lab, Uppsala University.

13.2 Simulating growth of 2D colonies on agar pad

13.2.1 Typical Phase Contrast Image Features in Images of Monolayer Colonies on Agar-pad

Phase contrast images of bacterial growth on agar pads are typically relatively low contrast, with additional texturing/patterning due to the characteristics of the solid agar medium on which the cells grow. Furthermore,

due to the interference effects from the phase contrast point spread function, the cells at the colony's perimeter are darker, surrounded by a bright halo at the interface between the medium and the cell. Another phase contrast artefact is the shade-off, which bleeds into the centre of the sample causing internal cells to be lighter in colour. While these artefacts are not easily seen in images of cells in linear colonies (e.g. mother machine images), they are very visible in almost all agar pad experiments. Our phase contrast image generation pipeline (described in Equation 7) was augmented with the addition of a very small offset to the PSF (dependent on the size of the kernel) which can be used to precisely modulate the amount of halo and shade-off in any given synthetic image. We found that a rough initial guess with random sampling around it was sufficient to generate very high quality training data capable of training highly accurate U-net models.

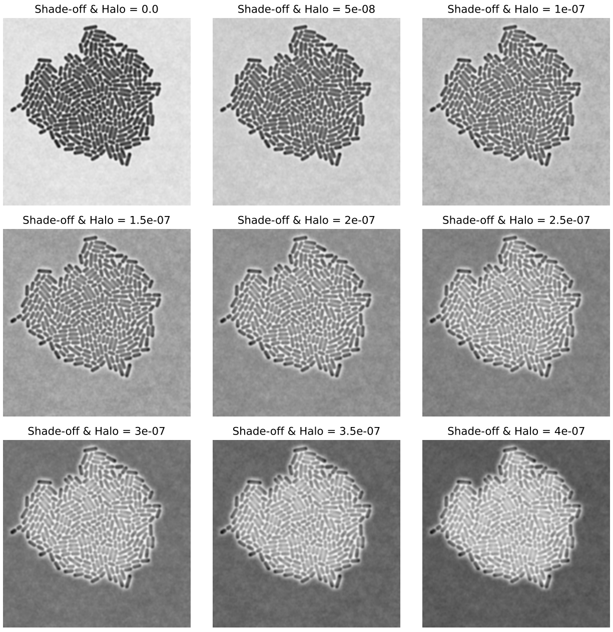

Figure S 13.2: Increasing shade-off and halo phase effects with increasing PSF offset.

Additionally, phase contrast agar pad images typically have some form of texturing in the background due to anisotropy in the agar and the pad drying over time. Perlin noise [13] is often used to generate natural looking surfaces and terrain height-maps in computer graphics. For this reason we used Perlin noise with a variety of randomised parameters to simulate the defects seen in phase contrast images of agar pads. Examples of this can be seen in Figure 13.3. The addition of this noise greatly increases training accuracy, as without it background noise is often positively segmented, creating spurious phantom masks.

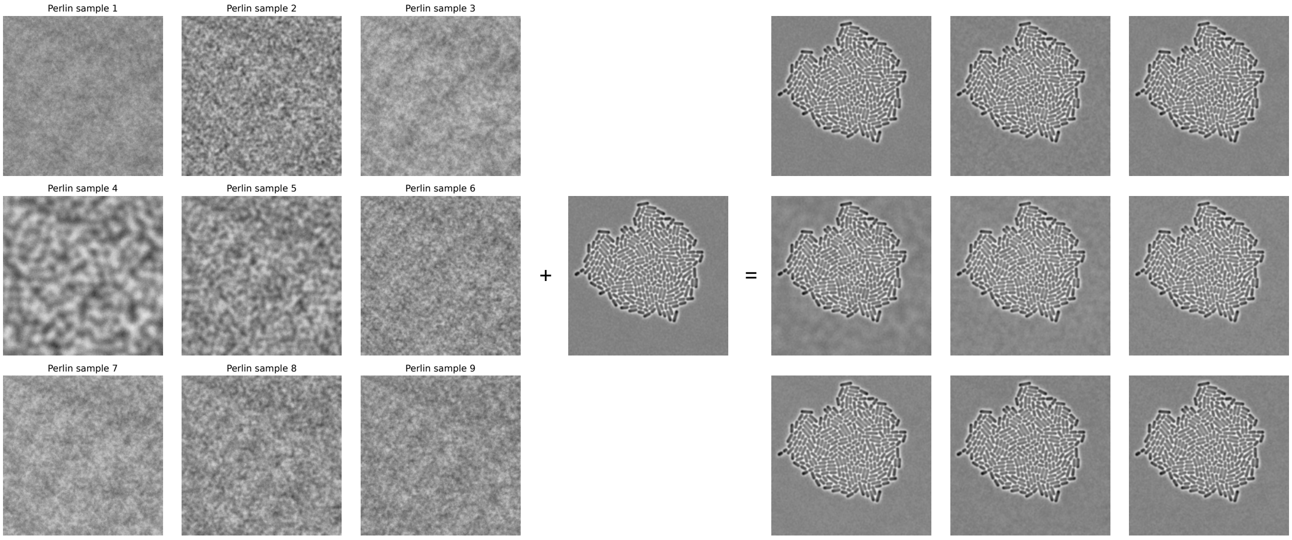

Figure S 13.3: Random sample of Perlin noise (shown on the left with enhanced contrast) made to mimic agar pad phase defects can be incorporated with synthetic images to create more realistic agar backgrounds.

To test the efficacy of this training data, we analysed bacteria growing in a monolayer on agar pads. An example montage of the segmentation is shown below. The performance on this data is very good considering no post-processing is done other than a simple threshold on the U-net probability output.

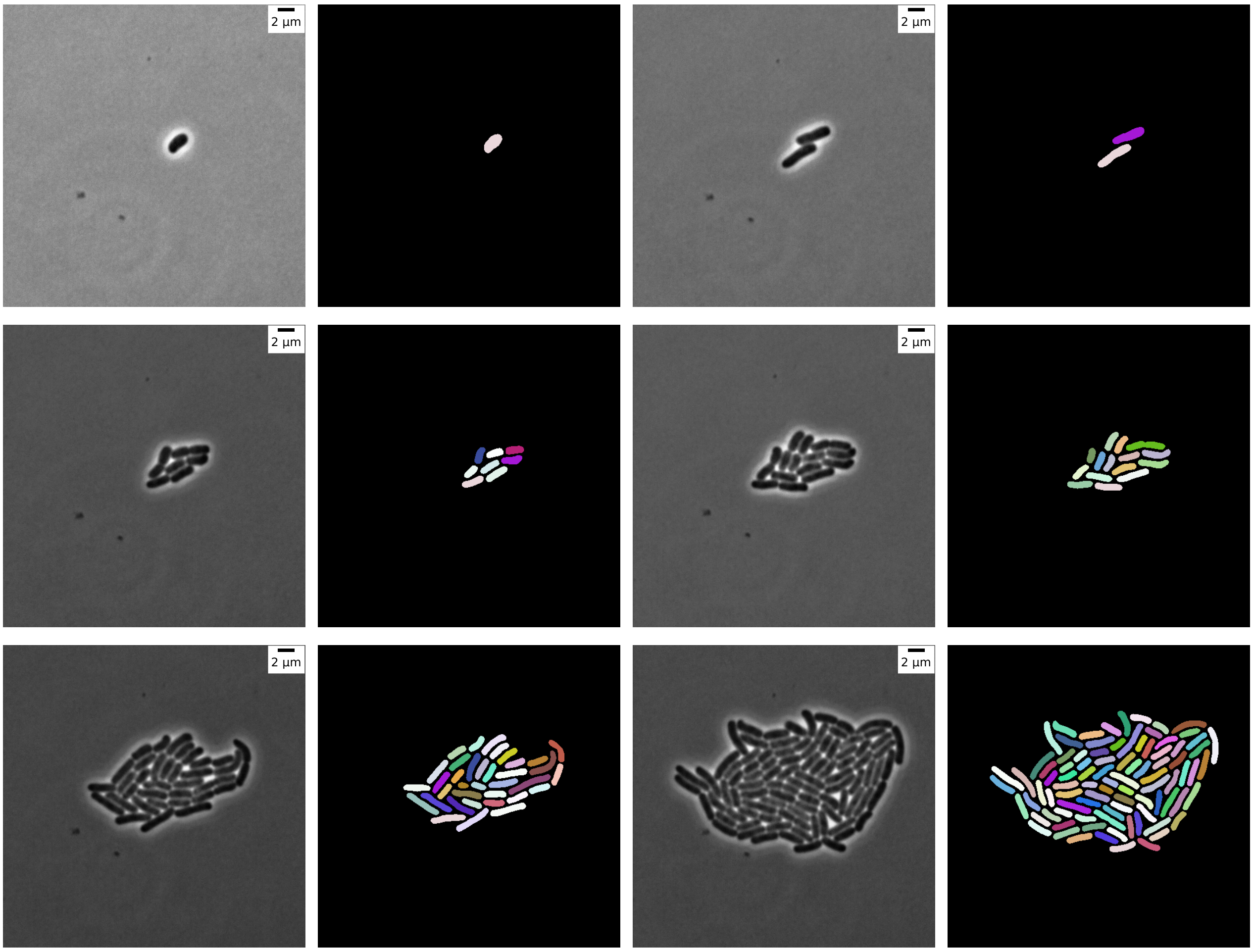

Figure S 13.4: Montage of *E. coli* growing on an agar pad, segmented using a U-net trained on SyMBac synthetic data.

13.2.2 Fluorescence Images of Monolayer Colonies on Agar Pad

Generating fluorescent data is almost identical to the generation of phase contrast data (described above), except we use a fluorescence PSF, and do not add any pseudorandom noise to the image background. The only noise added to the image is shot noise, which simulates the camera's noise (used in all image generation schemes). Additionally, a large variability was added in the brightness of each cell to account for real changes in fluorescent intensity due to stochastic gene expression. To test the ability of our synthetic data to train a model, we streaked a dense culture of stationary phase *E. coli* expressing YFP onto an agar pad and imaged a large field of view with a 60x air objective. These images were very large (1200x1200 pixels), and so synthetic training data of size 400x400 was generated (shown in Figure 13.5), and the image was segmented patch by patch. Examples of segmented FOVs are shown below in Figure 13.6.

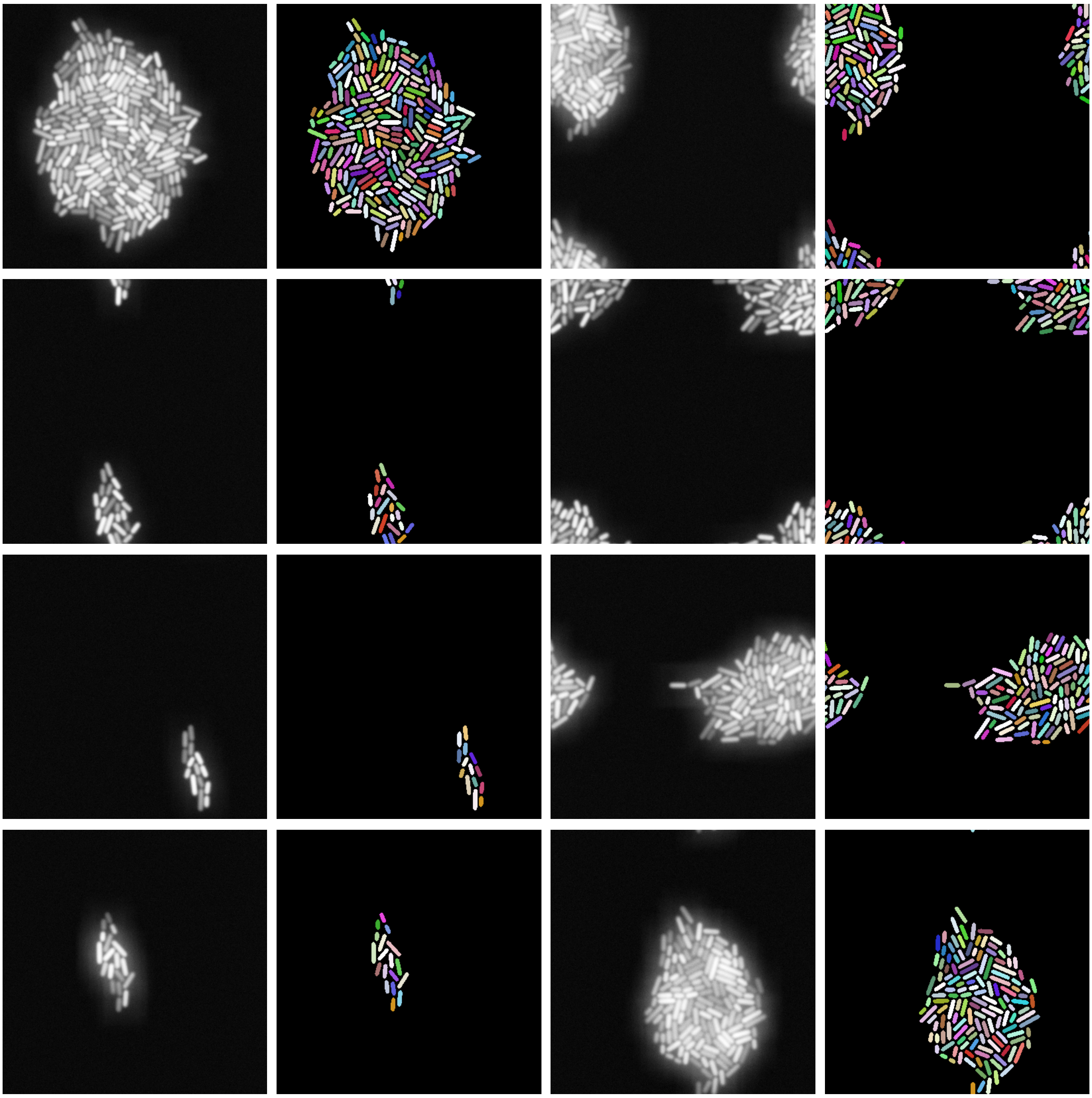

Figure S 13.5: Synthetic fluorescence training samples with accompanying masks.

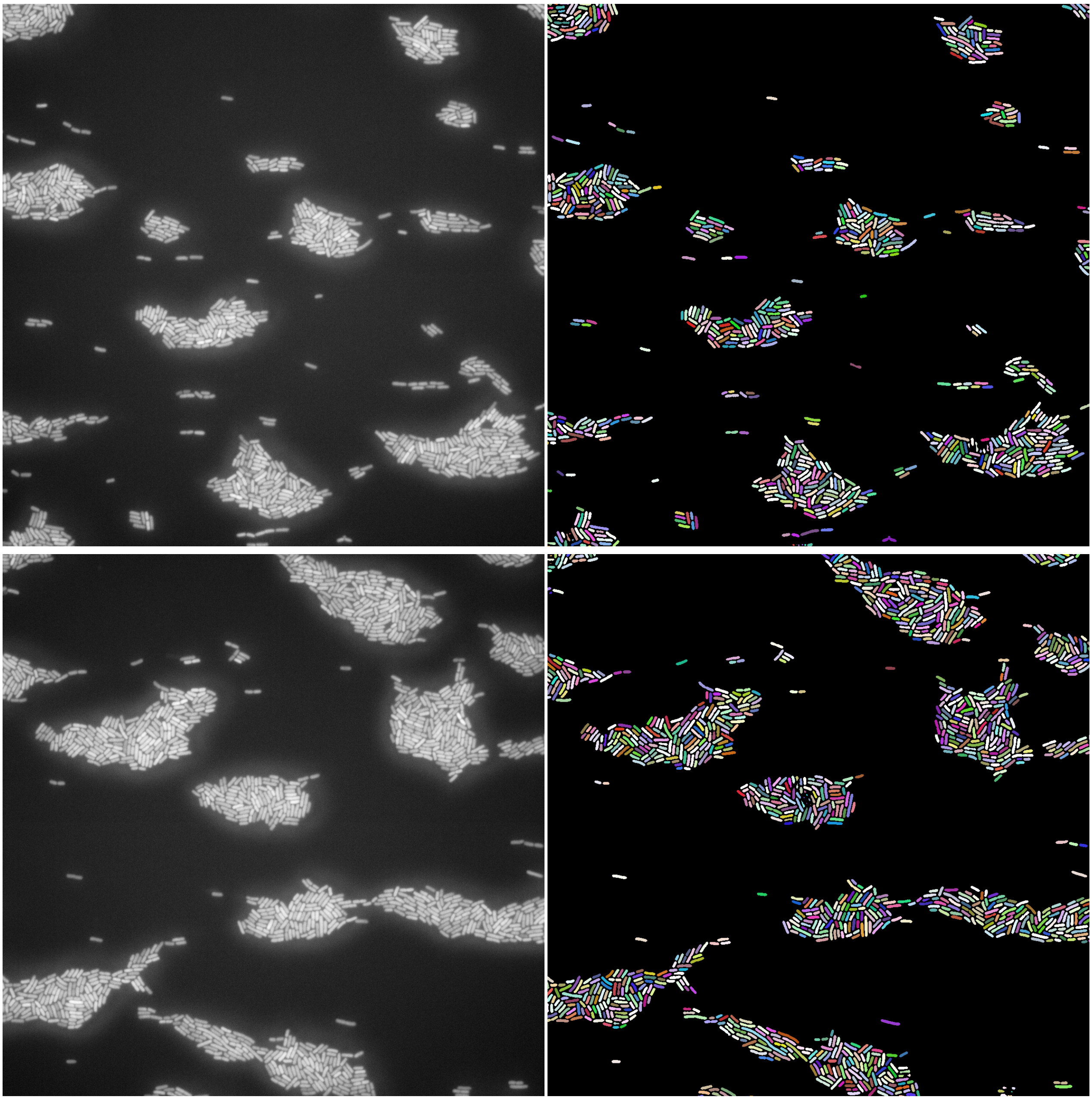

Figure S 13.6: Whole FOVs segmented patch by patch on a model trained on synthetic fluorescence data.

References

[1] Sofroniew, N., Lambert, T., Evans, K., Nunez-Iglesias, J., Winston, P., Bokota, G. *et al.* napari/napari: 0.4.10rc0 (2021). URL `https://doi.org/10.5281/zenodo.4968798`.

[2] Smit, J. H., Li, Y., Warszawik, E. M., Herrmann, A. & Cordes, T. Colicoords: A python package for the analysis of bacterial fluorescence microscopy data. *PLoS ONE* **14**, e0217524 (2019).

[3] Blomqvist, V. Pymunk: A easy-to-use pythonic rigid body 2d physics library (version 6.0.0) (2007). URL `https://www.pymunk.org`.

[4] Yin, Z., Kanade, T. & Chen, M. Understanding the phase contrast optics to restore artifact-free microscopy images for segmentation. *Medical Image Analysis* **16**, 1047–1062 (2012).

[5] Zhang, B., Zerubia, J. & Olivo-Marin, J. C. Gaussian approximations of fluorescence microscope point-spread function models. *Applied Optics, Vol. 46, Issue 10, pp. 1819-1829* **46**, 1819–1829 (2007).

[6] Mannan, F. & Langer, M. S. What is a good model for depth from defocus? *Proceedings - 2016 13th Conference on Computer and Robot Vision, CRV 2016* 273–280 (2016).

[7] Aguet, F., Geissbühler, S., Geissbühler, G., Märki, I., Lasser, T., Unser, M. *et al.* Super-resolution orientation estimation and localization of fluorescent dipoles using 3-d steerable filters. *Optics Express, Vol. 17, Issue 8, pp. 6829-6848* **17**, 6829–6848 (2009).

[8] Willenbockel, V., Sadr, J., Fiset, D., Horne, G. O., Gosselin, F. & Tanaka, J. W. Controlling low-level image properties: The shine toolbox. *Behavior Research Methods 2010 42:3* **42**, 671–684 (2010).

[9] van der Walt, S., Schönberger, J. L., Nunez-Iglesias, J., Boulogne, F., Warner, J. D., Yager, N. *et al.* scikit-image: image processing in python. *PeerJ* **2**, e453 (2014).

[10] Bakshi, S., Leoncini, E., Baker, C., Cañas-Duarte, S. J., Okumus, B. & Paulsson, J. Tracking bacterial lineages in complex and dynamic environments with applications for growth control and persistence. *Nature Microbiology 2021 6:6* **6**, 783–791 (2021).

[11] Rudge, T. J., Steiner, P. J., Phillips, A. & Haseloff, J. Computational modeling of synthetic microbial biofilms. *ACS Synthetic Biology* **1**, 345–352 (2012).

[12] Ullman, G., Wallden, M., Marklund, E. G., Mahmutovic, A., Razinkov, I. & Elf, J. High-throughput gene expression analysis at the level of single proteins using a microfluidic turbidostat and automated cell tracking. *Philosophical Transactions of the Royal Society B: Biological Sciences* **368** (2013).

[13] Perlin, K. An image synthesizer. *ACM SIGGRAPH Computer Graphics* **19**, 287–296 (1985).
